# Stalled disomes marked by Hel2-dependent ubiquitin chains undergo Ubp2/Ubp3-mediated deubiquitination upon translational run-off

**DOI:** 10.1038/s42003-025-07569-z

**Published:** 2025-01-28

**Authors:** Mario Scazzari, Ying Zhang, Anna Moddemann, Sabine Rospert

**Affiliations:** 1 Institute of Biochemistry and Molecular Biology, ZBMZ, Faculty of Medicine, University of Freiburg, Freiburg, Germany; 2 BIOSS Centre for Biological Signalling Studies, and CIBSS Centre for Integrative Biological Signalling Studies, University of Freiburg, Freiburg, Germany

**Keywords:** Ubiquitylated proteins, Ribosomal proteins, Ubiquitylation

## Abstract

Stalled ribosomes cause collisions, impair protein synthesis, and generate potentially harmful truncated polypeptides. Eukaryotic cells utilize the ribosome-associated quality control (RQC) and no-go mRNA decay (NGD) pathways to resolve these problems. In yeast, the E3 ubiquitin ligase Hel2 recognizes and polyubiquitinates disomes and trisomes at the 40S ribosomal protein Rps20/uS10, thereby priming ribosomes for further steps in the RQC/NGD pathways. Recent studies have revealed high concentrations of disomes and trisomes in unstressed cells, raising the question of whether and how Hel2 selects long-term stalled disomes and trisomes. This study presents quantitative analysis of in vivo-formed Hel2•ribosome complexes and the dynamics of Hel2-dependent Rps20 ubiquitination and Ubp2/Ubp3-dependent deubiquitination. Our findings show that Hel2 occupancy progressively increases from translating monosomes to disomes and trisomes. We demonstrate that disomes and trisomes with mono- or di-ubiquitinated Rps20 resolve independently of the RQC component Slh1, while those with tri- and tetra-ubiquitinated Rps20 do not. Based on the results, we propose a model in which Hel2 translates the duration of ribosome stalling into polyubiquitin chain length. This mechanism allows for the distinction between transient and long-term stalling, providing the RQC machinery with a means to select fatally stalled ribosomes over transiently stalled ones.

## Abstract (web summary)

The yeast E3 ligase Hel2 translates the duration of ribosome stalling into polyubiquitin chain length on Rps20, enabling the ribosome-associated quality control machinery to distinguish between transiently and fatally stalled ribosomes.

## Introduction

Ribosomal protein synthesis may come to a hold due to various factors, such as faulty transcripts, limiting concentration of charged tRNA, or nascent chains with arrest sequences^1–6^. In eukaryotic cells rescue of stalled ribosomes is mediated by the ribosome-associated quality control (RQC) pathway, which releases stalled ribosomes and initiates degradation of incomplete nascent chains and the no-go mRNA decay (NGD) pathway, which mediates endonucleolytic cleavage of improper transcripts^1–6^. The first non-ribosomal factor of the RQC and NGD pathways is the E3 ubiquitin ligase Hel2 (ZNF598 in mammals)^2–5^. Hel2 recognizes stalled ribosomes and attaches ubiquitin chains to 40S ribosomal proteins Rps20 (uS10), Rps10 (eS10), and Rps3 (uS3), which cluster near the ribosomal mRNA entry channel^7–15^. Matsuo et al.^10^ showed that mono- and di-ubiquitination of yeast Rps20 is critical for the initiation of the RQC and NGD pathways^10,14,16,17^. The major target of mammalian ZNF598 is Rps10 ^18^, however, recent evidence suggests that ubiquitination of Rps20 may also be a prerequisite for RQC in mammalian cells^13^. The yeast RQC-trigger (RQT) complex, and its mammalian counterpart *ASC-1*, bind to stalled ribosomes, which carry ubiquitinated Rps20^10,19^. The yeast RQT complex, comprised of the Ski2-like helicase Slh1 and the ubiquitin-binding proteins Cue3 and Rqt4^10,20^, mediates splitting of stalled ribosomes into 40S and 60S subunits, a prerequisite for downstream steps of the RQC/NGD pathways^10,13,14,16,17,19–22^.

Stalling of a single ribosome (termed leading ribosome) within a polysome causes collision with successive ribosomes (termed trailing ribosomes) and gives rise to structures termed disomes and trisomes^1–6^. The occurrence of disomes was first recognized in transcriptome-wide ribosome foot-printing experiments, because mRNA covered by disomes is protected from RNase digestion^23^. Later it was discovered that disomes constitute the substrate of the RQC and NGD pathways, suggesting that Hel2 recognizes and ubiquitinates disomes and trisomes^9^. Consistent with this prediction, formation of disomes and Rps20 ubiquitination was reconstituted in vitro with either strong stalling reporters, or supplementation of in vitro translation reactions with a dominant negative translation termination factor mutant^16,18^.

Moreover, the structures of mammalian^18^ and yeast disomes^16^, as well as yeast trisomes^17^ were solved by cryo-EM. Disomes display a unique structure, in which the leading ribosome adopts the non-rotated and the trailing ribosome adopts the rotated state. A hallmark of disomes generated in vitro is a stabilizing interface formed by the two 40S ribosomal Asc1 proteins (RACK1 in mammals) of the leading and trailing ribosomes and the above mentioned 40S ribosomal proteins targeted by Hel2 (termed Asc1-Asc1 platform)^16,18^. The interface between the second and third ribosome of trisomes resembles the interface of collided disomes^17^. Noteworthy, *HEL2*, *ASC1*, and the RQT-subunit *SLH1* display strong genetic interaction. Deletion/depletion of either gene blocks the RQC and NGD pathways and favors ribosomal read-through of stalling-prone transcript sites^4,8,12,20,21,24–26^. So far, structural information of Hel2 bound to disomes is unavailable, however, current models consider the Asc1-Asc1 platform as recognition motif, which allows Hel2 to specifically recognize disomes and trisomes^4,5^.

Ribosome stalling in vivo was mainly assessed in disome- and trisome-seq studies. These elegant approaches revealed that collisions are widespread on endogenous transcripts in yeast^23,27–29^, zebra fish^30^ and mice^31^. Zhao et al. estimated that in unstressed yeast cells about 6% of ribosomes form disomes^28^. A meta-analysis performed by Diament and coworkers revealed that up to 20% of ribosomes form RNase-resistant disomes^27^. Accordingly, a yeast cell, which contains about 300,000 ribosomes^32^, harbors an estimated number of 9000–30,000 disomes^27,28^_,_. In comparison, the components of the RQC machinery are expressed at a rather low level^24,33^. As an example, a yeast cell contains about 3000 molecules of Hel2^34^. Moreover, the process of RQC is slow when compared to translation elongation and termination^35^. The above observations raise the question if the RQC pathway indeed accomplishes the resolution of all disomes, or rather targets only a subset. In support of the latter, RNase-resistant disomes generated in vitro^16,18^ turned out to be structurally different from the bulk of RNase-resistant disomes in log-phase cells^28^. In vivo, the leading ribosome of disomes is in a rotated state and the interface between the two ribosomes is flexible, without close contacts between the two Asc1 proteins^28^. To complete the picture, more recent cryo-EM studies reveal that disomes can adopt a variety of structures, depending e.g. on the trigger for stalling^3,13,14,28,36^.

Available disome/trisome-seq studies do not provide information about ribosome-binding of Hel2 or the ubiquitination status of Rps20^23,27–29^. Sucrose gradient sedimentation demonstrated that Hel2 comigrates with polysomes in unstressed yeast cells^10,37^. However, polysomes contain only few disomes/trisomes, and in vivo crosslinking demonstrated that Hel2 may also bind to monosomes^37^. Data obtained in mammalian cells cannot be generalized because ZNF598, unlike Hel2, is cytosolic^8,11,18^ and shifts to polysomes only after induction of stalling^18^. In vitro translation/ubiquitination approaches employed excess purified Hel2 and focused on Rps20 ubiquitination; however, did not address Hel2 binding to ribosomal complexes directly^10,14,16,17^. Thus, despite significant progress in our understanding of the RQC/NGD pathways, the initial selection of ribosomal complexes by Hel2 and the dynamics of Rps20 ubiquitination/deubiquitination in living cells remain poorly understood.

In this study, we present a comprehensive analysis of in vivo-formed Hel2•ribosome complexes and the dynamics of Rps20 ubiquitination/deubiquitination under various growth conditions and in relevant mutant strains. Our findings reveal that Hel2 forms salt-sensitive complexes with translating ribosomes, disomes, and trisomes in log-phase cells. When ribosome stalling is induced, the concentration of disomes, and even more so trisomes, rises and Hel2 forms salt-resistant complexes. We show that log-phase cells contain a steady concentration of Rps20 species attached to up to four ubiquitin moieties. When ribosome stalling is induced, the concentration of disomes and trisomes, the concentration of ubiquitinated Rps20, and the length of polyubiquitin chains attached to Rps20 increase within minutes. Conversely, when translational run-off is induced by glucose depletion, the concentration of disomes/trisomes and Rps20 ubiquitination quickly drops to a low level. We identify Ubp2 and Ubp3 as deubiquitinases of Rps20 and demonstrate that they mediate efficient deubiquitination of Rps20 upon translational run-off. Deubiquitination of mono- and di-ubiquitinated Rps20 species remained largely unaffected in a strain lacking the RQT-subunit Slh1. Tri- and tetra-ubiquitinated Rps20 species, however, were significantly stabilized in the absence of Slh1. Based on the combined data, we propose a model wherein the dwell time of disomes and trisomes determines the number of ubiquitin moieties Hel2 attaches to Rps20. In this model, disomes that briefly pause before resuming translation carry only one or two ubiquitin moieties. Conversely, more severely stalled disomes, marked by longer polyubiquitin chains, require resolution through the RQC pathway.

## Results

### Hel2 is associated with translating ribosomes and collided disomes

**Fig. 1:**
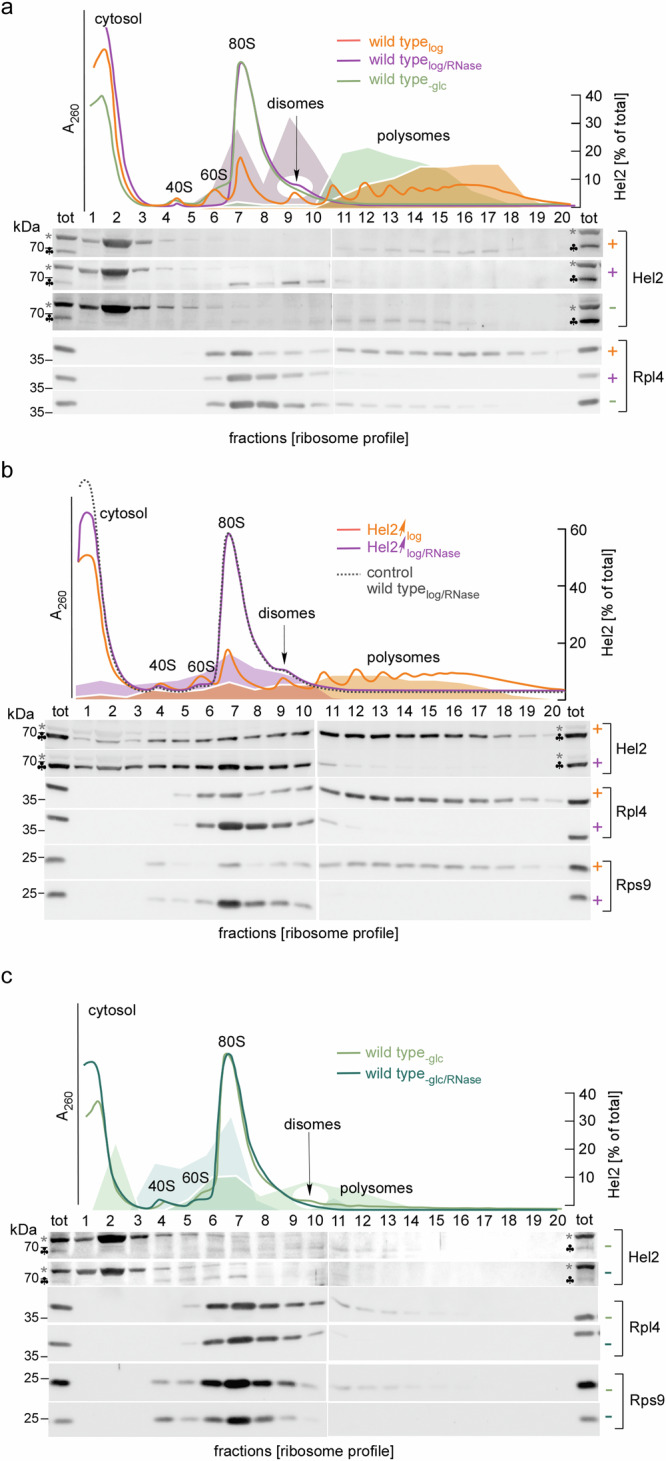
Hel2 is associated with translating ribosomes and collided disomes. **a** Hel2 binds to translating ribosomes and collided disomes. Polysome profiles were generated by sucrose gradient sedimentation (see Methods) with untreated log-phase extract (wild type_log_, orange), after RNase treatment (wild type_log/RNase_, magenta), or with extract derived from glucose depleted cells (wild type_-glc_, light green). The approach is detailed in Fig. S1a. **b** Upon overexpression, a large fraction of Hel2 is bound to translating ribosomes. Polysome profiles with log-phase extract from Hel2↑ cells without (Hel2↑_log_, orange) or after RNase treatment (Hel2↑_log/RNase_, magenta). For immunodetection of overexpressed Hel2, loading was reduced to 1/3 of the wild type. An A_260_ trace of wild type_log/RNase_ (dotted gray line), recorded side by side, is shown as a control. **c** Hel2 does not bind to non-translating ribosomes. Cells were starved for glucose and subsequently sucrose density sedimentation was performed with untreated extract (wild type_-glc_, light green) and after RNase treatment (wild type_-glc/RNase_, dark green). **a**–**c** A_260_ profiles, Hel2 immunoblots, Hel2 distribution profiles (see Methods), and immunoblots of the 60S ribosomal protein Rpl4 and the 40S ribosomal protein Rps9. The total (tot) corresponds to 5% of the extract loaded onto the gradient. The position of 40S, 60S, 80S, disomes, and higher order polysomes is indicated. Clubs indicate Hel2 bands, gray asterisks indicate a background band recognized by α-Hel2 (Fig. S1b). The disome region of A_260_ traces is marked by a white spotlight.

To better understand the initiation phase of the RQC/NGD pathways, we analyzed Hel2 binding to ribosomal particles by sucrose gradient density centrifugation (see Methods). For that purpose, we prepared yeast extract from cells grown under well-defined physiological conditions (Fig. S1a). In glucose-grown log-phase cells (termed wild type_log_) most ribosomes formed polysomes to which, consistent with previous reports^10,37^, the bulk of Hel2 was bound (Fig. 1a, orange A_260_ trace, Rpl4 and Hel2 immunoblots, and Hel2 distribution profile and Fig. S1b). Polysomes mostly represent non-stalled, actively translating ribosomes, however, may contain also disomes and trisomes_._ It thus remained unclear to which type of ribosomal particle Hel2 was bound. To address this question, wild type_log_ extract was treated with RNase (termed wild type_log/RNase_). The treatment converts non-stalled polysomal ribosomes into 80S monosomes, attached to peptidyl-tRNA (termed translating monosomes), however, leaves collided disomes and trisomes intact (Fig. S1a). Wild type_log/RNase_ contained mainly translating monosomes and a low amount of RNase-resistant disomes, which formed a shoulder overlapping with the major translating monosome peak (Fig. 1a, magenta A_260_ trace and Rpl4 immunoblot). Please note that the disome peak in untreated wild type_log_ extract (Fig. 1a, orange A_260_ profile) mainly consists of two non-collided ribosomes associated with a single transcript, which is digested, and converted to translating monosomes by RNase. In contrast, the disome peak in wild type_log/RNase_ extract (Fig. 1a, magenta A_260_ profile) is mainly derived from polysomal disomes (Fig. S1a). Hel2 was distributed between translating monosome and disome fractions in wild type_log/RNase_ extract (Fig. 1a, magenta Hel2 immunoblot and Hel2 distribution profile). Because translating monosomes were in vast excess over RNase-resistant disomes (Fig. 1a, magenta A_260_ trace) the distribution of Hel2 suggested that the apparent affinity (affinity_app_, see Methods) of Hel2 for disomes was significantly higher when compared to translating monosomes. However, ribosome-binding of Hel2 was not confined to disomes (see below).

We generated a Hel2↑ strain overexpressing Hel2 approximately 30-fold (Fig. S1c). In this strain Hel2 was more abundant than disomes and trisomes (Hel2↑ cell: ~90,000 (30 × 3000) molecules of Hel2; for disome/trisome abundance see Introduction and below). The concentration of RNase-resistant disomes in the Hel2↑ strain resembled the wild type indicating that Hel2 overexpression did not induce ribosome stalling (Fig. 1b, magenta and dotted gray A_260_ traces). Overexpressed Hel2 was mainly associated with polysomes (Fig. 1b, orange A_260_ trace and Hel2 distribution profile). After RNase treatment Hel2 was mainly associated with translating monosomes, smearing into lower molecular mass fractions, suggesting gradual release of Hel2 from translating monosomes (Fig. 1b, magenta A_260_ trace, Hel2 immunoblot and distribution profile). We conclude that the bulk of Hel2 was associated with translating monosomes when Hel2 was expressed in excess relative to RNase-resistant disomes/trisomes (Fig. 1a, b).

With the exception of few transcripts essential for metabolic adjustments, yeast rapidly shuts off translation initiation when glucose runs short^38–40^. Hence, most ribosomes complete a last round of translation (termed translational run-off) and turn into non-translating monosomes (Fig. S1a). Sucrose gradient sedimentation was performed with extract derived from 10 min glucose depleted cells (termed wild type_-glc_). As expected, only few polysomes, detectable with the sensitive Rpl4 antibody, survived glucose depletion (Fig. 1a, light green Rpl4 immunoblot, compare to magenta wild type_log/RNase_ Rpl4 immunoblot). The 80S peaks of wild type_-glc_ (non-translating monosomes) and wild type_log/RNase_ (translating monosomes) were nearly congruent. However, the disome shoulder was only present in the wild type_log/RNase_ profile (Fig. 1a, magenta and light green A_260_ traces and Fig. S1d). Thus, the bulk of disomes had completed one last round of translation minutes after glucose was removed from the medium. Even though the concentration of polysomal ribosomes in wild type_-glc_ was low, Hel2 was mostly associated with these residual polysomes (Fig. 1a, c, light green A_260_ traces and Hel2 distribution profile). Upon treatment with RNase (wild type_-glc/RNase_), disomes were below the detection limit (Fig. 1c, light green and dark green Rpl4 and Rps9 immunoblots). Consistent with the absence of disomes, Hel2 was recovered in the 80S fractions of the wild type_-glc/RNase_ profile, smearing into lower molecular mass fractions (Fig. 1c, dark green Hel2 distribution profile).

### Ribosome collision induces salt-resistant binding of Hel2 to disomes

**Fig. 2:**
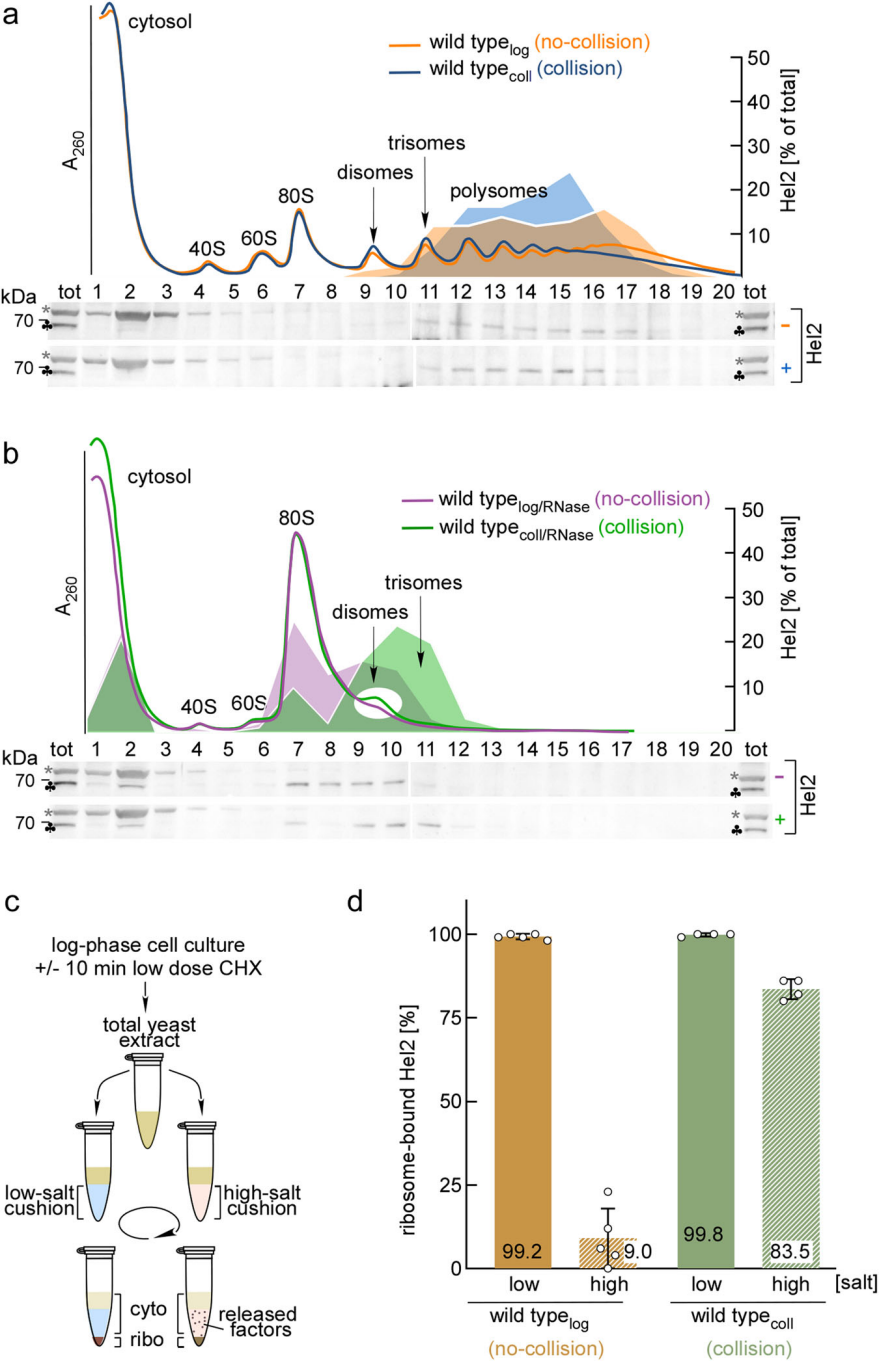
Induction of ribosome collision with low-dose CHX induces salt-resistant binding of Hel2. **a** Hel2 binds to polysomes with or without induction of ribosome collision. Polysome profiles with extract derived from wild type_log_ (orange) or with 10 min low-dose CHX-treated wild type_coll_ (blue). **b** The bulk of Hel2 binds to disomes and trisomes after induction of ribosome collision. Wild type_log_ and wild type_coll_ as shown in (**a**) after RNase treatment. A_260_ profiles, Hel2 immunoblots, and Hel2 distribution profiles of wild type_log/RNase_ (magenta) and wild type_coll/RNase_ (green). The disome region of the A_260_ trace is marked by a white spotlight. Polysome profiles in (**a**, **b**) are from the same experiment. The total (tot) corresponds to 5% of the extract loaded onto the gradient. Labeling is as detailed in Fig. 1. **c** Ribosome-binding assay. To determine the stability of Hel2•ribosome complexes extracts were loaded onto sucrose cushions and ribosomes (ribo) were collected by ultra centrifugation. The ribosomal pellet contains polysomes, monosomes, and ribosomal subunits. Smaller proteins/complexes, such as unbound Hel2, remain in the supernatant. When the cushion contains low salt (120 mM KAc) ribosome-associated factors co-sediment with ribosomes. When the cushion contains high salt (800 mM KAc) only core ribosomal proteins are recovered in the ribosomal pellet. **d** Induction of ribosome collision induces salt-resistant binding of Hel2. Ribosome-binding of Hel2 was assessed in wild type_log_ (no-collision, beige) or wild type_coll_ (collision, green) extract under low- or high salt conditions. Shown is the fraction of Hel2 co-sedimenting with ribosomes in at least 4 independent experiments (bars) and the result of each experiment (dots) with the standard deviation. An example blot is shown in Fig. S2.

Low concentrations of translational inhibitors such as cycloheximide (CHX) induce disomes, because uninhibited trailing ribosomes collide with stalled ribosomes bound to CHX^4,9,18,28,41,42^. To test the effect of induced ribosome stalling, extract was prepared from log-phase cells after 10 min low-dose (0.5–1 µg/ml) CHX treatment (wild type_coll_). The distribution of ribosomes and Hel2 in wild type_coll_ was similar to untreated wild type_log_ (Fig. 2a, orange and blue A_260_ traces and Hel2 distribution profiles). However, after RNase treatment it became clear that the disome concentration was increased after low-dose CHX treatment (Fig. 2b, compare magenta and green A_260_ traces). Moreover, Hel2 was shifted from translating monosomes (Fig. 2b, magenta Hel2 immunoblots and Hel2 distribution profiles) to disome, and even trisome fractions (Fig. 2b, green Hel2 immunoblots and Hel2 distribution profiles). The data confirmed that Hel2 was able to bind to translating monosomes, however, the affinity_app_ of Hel2 for disomes and trisomes was significantly higher (see below).

Most disomes isolated from log-phase cells do not contain the typical Asc1/Asc1 platform; however, in vivo CHX-treatment induces a disome structure, that resembles in vitro-generated disomes^28^ (see Introduction). To test if induction of ribosome stalling affected the interaction of Hel2 with ribosomal complexes, we employed a well-established ribosome-binding assay, which distinguishes between salt-sensitive and salt-resistant binding of ribosome-associated factors (Fig. 2c^43,44^,). In low salt conditions, Hel2 was fully ribosome-bound in both in wild type_log_ and wild type_coll_ extract (Figs. 2d and  S2). In high salt conditions, however, less than 10% of Hel2 was ribosome-bound in wild type_log_, while more than 80% of Hel2 was ribosome-bound in wild type_coll_ extract (Figs. 2d and S2). Thus, induction of collision not only raised the concentration of disomes (Fig. 2b, green A_260_ trace), but also induced salt-resistant binding of Hel2 (Fig. 2d). The data suggested that Hel2 binds in a salt-sensitive manner to translating monosomes and disomes, which predominate in log-phase cells and lack the Asc1-Asc1 platform^28^, whereas Hel2 binds in a salt-resistant manner to disomes/trisomes, which accumulate under stalling conditions and contain the Asc1-Asc1 platform^16,18,28^ (see also below).

### Catalytically inactive Hel2-RM binds salt-resistantly to disomes and trisomes

**Fig. 3:**
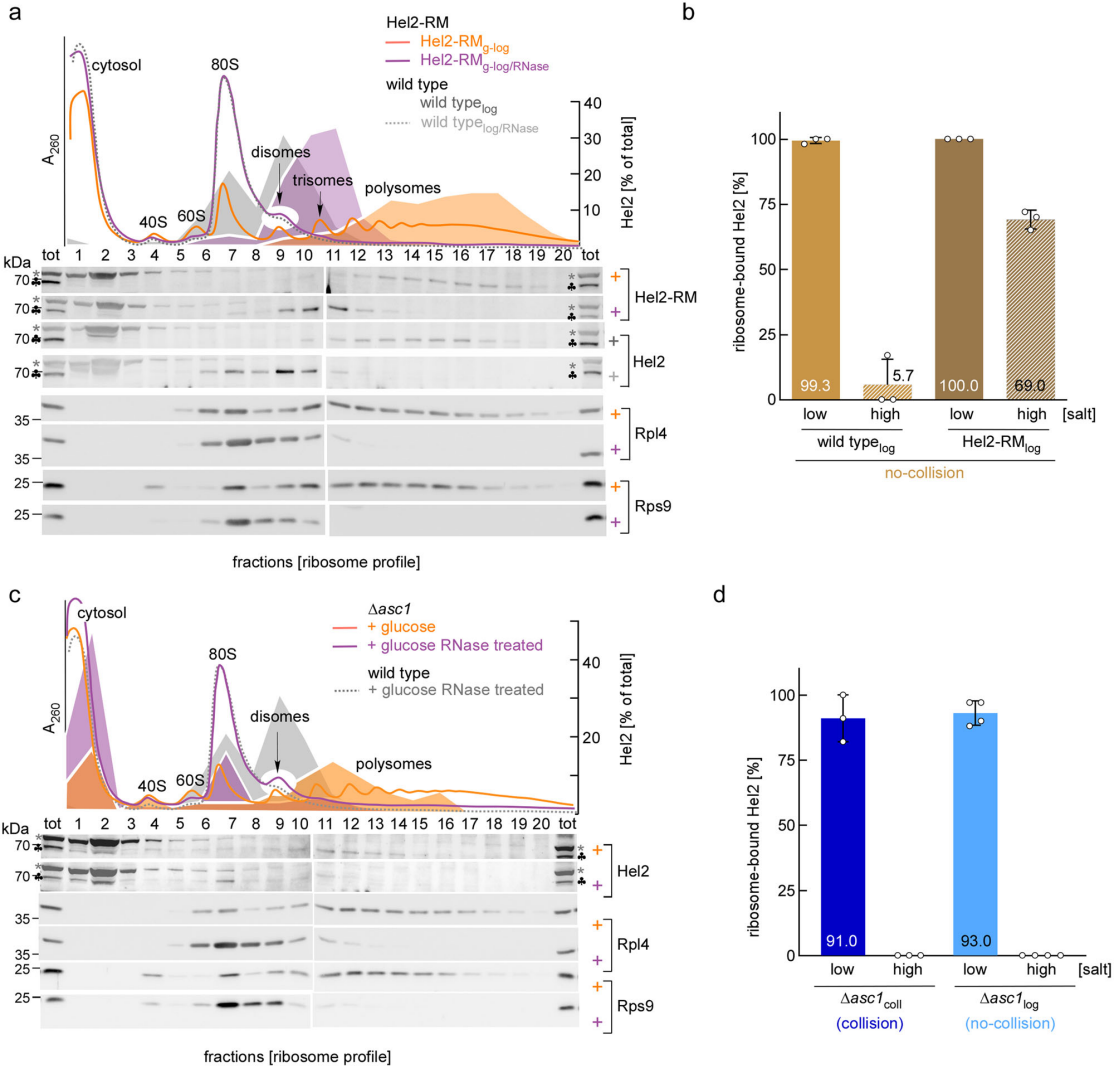
Collided disomes accumulate in cells expressing catalytically inactive Hel2-RM or lacking the 40S ribosomal protein Asc1. **a** The bulk of Hel2-RM binds to disomes and trisomes Polysome profiles with extracts derived from Hel2-RM_g-log_ (orange) or Hel2-RM_g-log/RNase_ (magenta). **b** Hel2-RM binds to ribosomes in a salt-resistant manner. Ribosome binding assays with extract of wild type_log_ or Hel2-RM_log_ as described in Fig. 2c. Shown is the mean of 3 independent experiments (bars) and the result of each experiment (dots) with the standard deviation. An example blot is shown in Fig. S3a. **c** High affinity binding of Hel2 to disomes depends on Asc1. Polysome profiles with extracts of ∆*asc1*_log_ (orange) or ∆*asc1*_log/RNase_ (magenta). **d** Hel2 binds to ∆*asc1-*disomes in a salt-sensitive manner, with or without induction of collision. Ribosome-binding assays were performed with extract of ∆*asc1*_log_ (no-collision, blue) or ∆*asc1*_coll_ (collision, light blue). Shown is the mean of at least 3 independent experiments (bars) and the result of each experiment (dots). An example blot is shown in Fig. S3d. Polysome profiles shown in **a** and **c**, including wild type_log/RNase_ A_260_ traces (dotted gray lines) and Hel2 distribution profiles of wild type_log/RNase_ (gray), were recorded side by side. Labeling is as detailed in Fig. 1.

Resolution of stalled ribosomes requires the catalytic activity of Hel2 (^4,10,16^, see Introduction). We thus expected that the concentration of disomes, to which Hel2 binds salt-resistantly, should be increased in cells expressing catalytically inactive Hel2. A single cysteine mutation (C64S) was genomically introduced into the **R**ING **D**omain of Hel2 (termed Hel2-RM_g_, see also below). The distribution of Hel2-RM_g_ in polysome profiles resembled wild type Hel2 (Fig. 3a, orange A_260_ trace and orange and dark gray Hel2/Hel2-RM distribution profiles). However, the concentration of RNase-resistant disomes in Hel2-RM_g-log/RNase_ extract was increased when compared to wild type_log/RNase_ extract (Fig. 3a, compare magenta and dotted gray A_260_ traces). Moreover, Hel2-RM was shifted to disome and trisome fractions when compared to Hel2 (Fig. 3a, magenta and light gray Hel2/Hel2-RM immunoblots and distribution profiles). In contrast to wild type Hel2, the bulk of Hel2-RM was salt-resistantly bound, even without induction of collision (Figs. 3b and  S3a–c). The findings are consistent with a model in which cells expressing catalytically inactive Hel2-RM, due to the well-established disruption of the RQC pathway^10,14,16,17^, accumulate disomes/trisomes, to which Hel2-RM binds in a salt-resistant manner.

### The 40S ribosomal protein Asc1 is required for recruitment and salt-resistant binding of Hel2 to disomes and trisomes

To further test if the Asc1/Asc1 platform was involved in salt-resistant binding of Hel2, we employed a ∆*asc1* strain. Consistent with published data^37^, Hel2 was mainly recovered in polysomal fractions of ∆*asc1*_log_ extract, however, the distribution of Hel2 was shifted to lower order polysomes and Hel2 smeared into 80S and cytosolic fractions (Fig. 3c, orange immunoblot and Hel2 distribution profile). Interestingly, this was not due to a shortage of disomes. Rather, the concentration of RNase-resistant disomes in ∆*asc1* cells was higher compared to wild type (Fig. 3c, magenta and dotted gray A_260_ traces). Hel2, however, was not associated with RNase-resistant ∆*asc1*-disomes/trisomes, but only with translating ∆*asc1*-monosomes (Fig. 3c, magenta Hel2 immunoblot and distribution profile). RNase-treatment further reduced the fraction of Hel2 associated with ∆*asc1*-ribosomes (Fig. 3c, magenta Hel2 immunoblot and distribution profile). This is consistent with previous findings, which revealed that Hel2 binding to ribosomes is in part mediated by mRNA, which is digested by RNase^37^. Hel2 was associated with ∆*asc1*-ribosomes in low salt conditions, however, binding was salt-sensitive, and remained fully salt-sensitive even after induction of collision (Figs. 3d and  S3d). The data indicate that Asc1 was neither required for ribosome stalling, nor for the binding of Hel2 to translating monosomes. However, Asc1 was required for salt-resistant binding of Hel2 to disomes/trisomes. This is consistent with a model in which salt-resistant binding of Hel2 depends on the formation of the Asc1-Asc1 platform (see Discussion).

### Assessment of Hel2-ribosome complexes

**Fig. 4:**
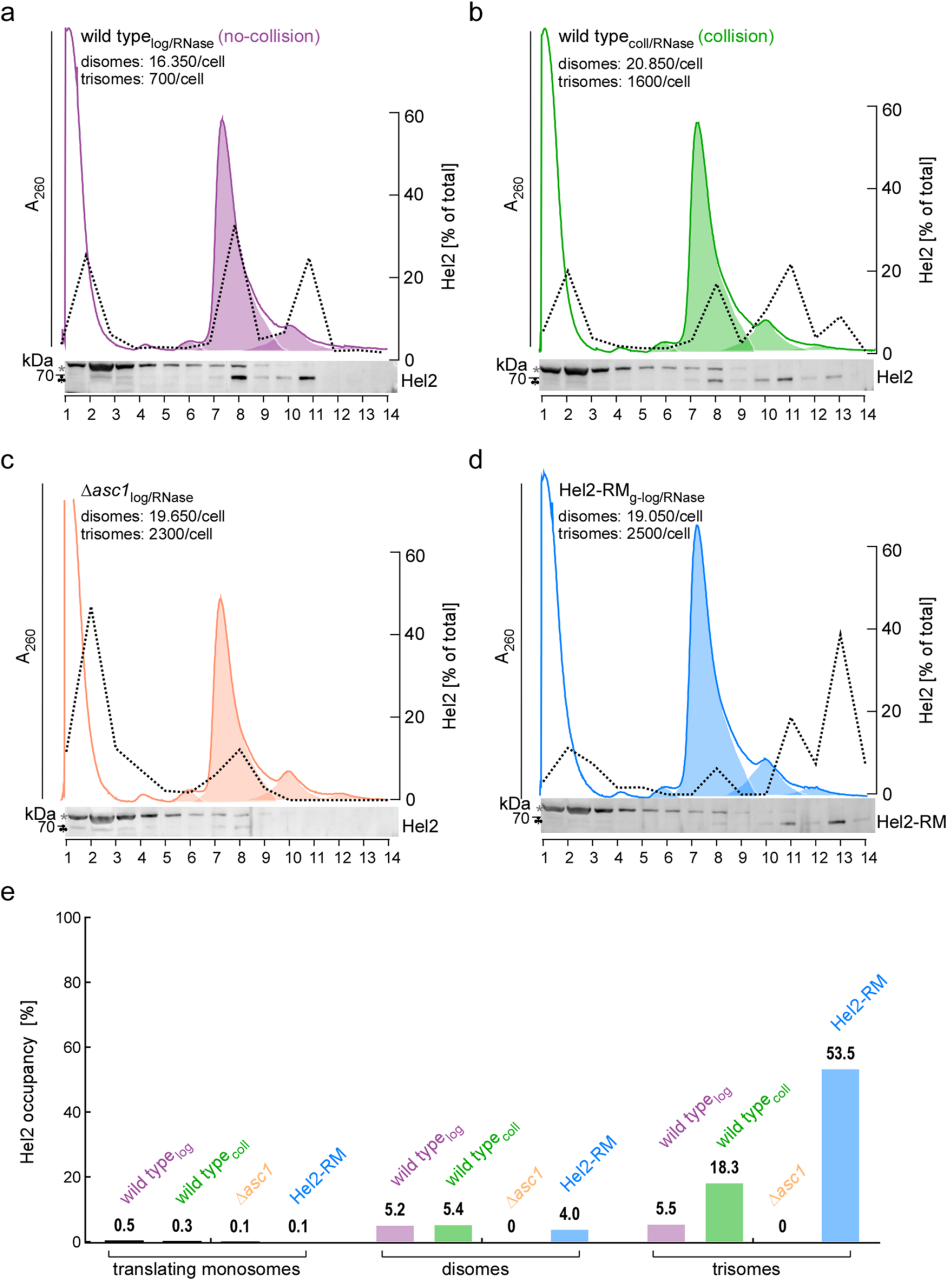
Hel2 occupancy on ribosomal complexes. Polysome profiles and Hel2 distribution profiles of RNase-treated extracts were recorded side by side (see Methods). To avoid overloading of immunoblots, loading of fraction 2 was reduced to 50%. Peak deconvolution and quantification of monosomes, disomes, and trisomes is described in Methods. Estimated numbers of disomes and trisomes per cell are based on the assumption that a yeast cell harbors approximately 300,000 ribosomes^32^. Shown are A_260_ profiles (colored solid lines), Hel2 immunoblots, Hel2 distribution profiles (dotted black lines), and the distribution of monosomes, disomes, and trisomes obtained by peak deconvolution (colored areas under the A_260_ profiles) of (**a**) wild type_log/RNase_ (**b**) wild type_coll/RNase_ (**c**) ∆*asc1*_log/RNase_ (**d**) Hel2-RM_g-log/RNase_. **e** Hel2 occupancy on translating monosomes, disomes, and trisomes. The percentage of total Hel2 (approximately 3000 molecules/cell^34^) associated with monosomes (fractions 7–9), disomes (fractions 10–12), and trisomes (fractions 13 and 14) was calculated as described in Methods and Supplementary Data 1.

To gain further insight into the concentration of ribosomal complexes and their occupancy by Hel2, we optimized polysome profiling for the analysis and quantification of monosomes, disomes, and trisomes in RNase-treated extracts (see Methods and Supplementary Data 1). The analysis revealed that wild type cells (wild type_log/RNase_) contained ~ 16,000 RNase-resistant disomes and 700 trisomes, corresponding to ~ 11% and ~ 0.7% of total ribosomes, respectively (Fig. 4a). These numbers align well with previous disome/trisome-seq studies^23,27–29^. Compared to wild type_log/RNase_, the concentration of disomes increased only moderately by about 25% after 10 min low-dose CHX treatment (wild type_coll/RNase_, Fig. 4b), in cells lacking Asc1 (∆*asc1*_log/RNase_, Fig. 4c), or in cells expressing catalytically inactive Hel2-RM (Hel2-RM_log/RNase_, Fig. 4d). In contrast, the concentration of trisomes increased more than two-fold upon low-dose CHX treatment (wild type_coll/RNase_, Fig. 4b), and more than 3-fold in ∆*asc1* (∆*asc1*_log/RNase_, Fig. 4c) or Hel2-RM strains (Hel2-RM_log/RNase_, Fig. 4d). Thus, overloading (low-dose CHX treatment) or impairment (∆*asc1* or Hel2-RM strains) of the RQC pathway exerted little effect on the steady state disome content but caused significant accumulation of trisomes.

Hel2 occupancy on ribosomal complexes was estimated (see Methods and Supplementary Data 1) assuming that a log-phase yeast cell contains 300,000 ribosomes^32^ and 3000 molecules of Hel2^34^. Hel2 occupancy on translating monosomes was between 0.1 to 0.5% (Fig. 4e). Hel2 occupancy on disomes ranged between 4.0 and 5.4% (Fig. 4a, b, d, e), with the exception of the ∆*asc1* strain, in which Hel2 did not associate with disomes or trisomes (Figs. 3c and 4c, e). In unstressed wild type cells Hel2 occupancy on disomes (5.2%) and trisomes (5.5%) (Fig. 4a, e) was very similar. However, upon low-dose CHX treatment Hel2 occupancy on trisomes was above 18% (Fig. 4b, e), and more than 50% of trisomes were occupied with Hel2-RM (Fig. 4d, e). The results reveal that Hel2 occupancy increases progressively from translating monosomes to disomes and trisomes (see also Discussion).

### Proteasomal degradation of Hel2 is enhanced in the absence of Asc1

**Fig. 5:**
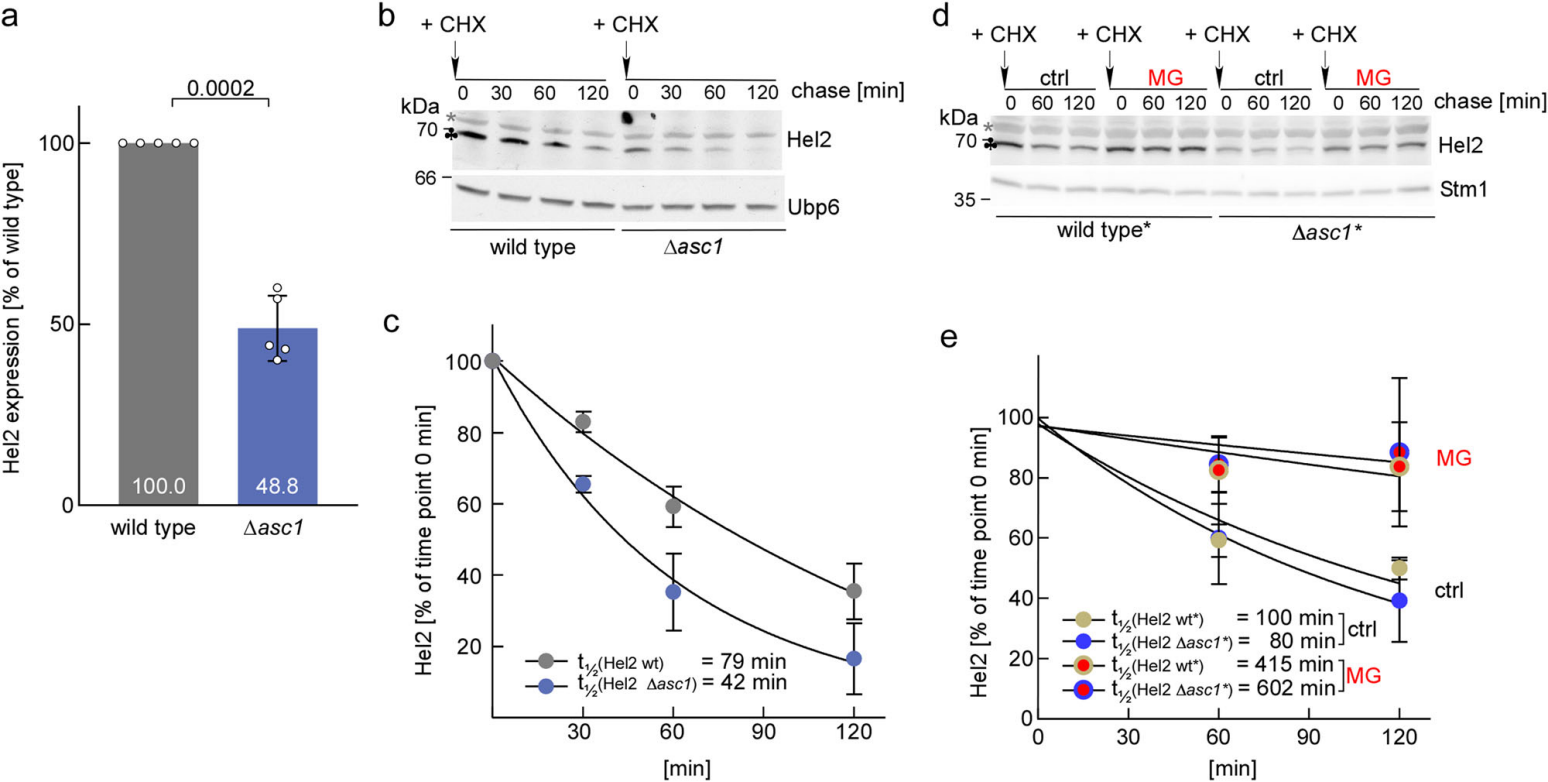
Proteasomal degradation of Hel2 is enhanced in the absence of Asc1. **a** Steady state expression of Hel2 is reduced in ∆*asc1* cells. The Hel2 expression level in wild type or ∆*asc1* lysate was analyzed by immunoblotting with α-Hel2. An example blot is shown in Fig. S5. For each replicate the intensity of the Hel2 band in the wild type lysate was set to 100%. Shown is the mean of 5 experiments and the result of each experiment (dots) with the standard deviation and the result of a paired samples t-test. **b** Turnover of Hel2 in wild type and ∆*asc1* cells. After addition of high-dose CHX aliquots were withdrawn from cell cultures and were analyzed by immunoblotting with ∝-Hel2 and α-Ubp6 (loading control). For details see Methods. **c** Exponential decay fit of CHX-chase repeats as shown in **b**. Shown is the mean of four independent experiments. Band intensities were normalized to time point 0 of wild type or ∆*asc1*, respectively. The average half life (t½) of Hel2 in wild type (gray) and ∆*asc1* (light blue) is indicated. **d** Inhibition of the proteasome with MG132 stabilizes Hel2. CHX-chase experiments were performed with wild type* (∆*erg6*) and ∆asc1* (∆*asc1*∆*erg6*) strains mock-treated (ctrl) or pre-treated with MG132 (MG) as described in Methods. At the time points indicated, aliquots were withdrawn and were analyzed by immunoblotting with α-Hel2 and α-Stm1 (loading control). **e** Exponential decay fit of four independent CHX chase experiments as shown in **d**. Band intensities were normalized to the time point 0 of each replicate. The average half life (t½) of Hel2 in wild type* (beige) and ∆asc1* (dark blue) is indicated. Samples pre-treated with MG132 (MG) are labeled in red.

In the course of the above experiments, we observed that the steady state level of Hel2 in the ∆*asc1* strain was about two-fold lower when compared to wild type (Figs. 5a and Fig. S5). As the expression of *HEL2* in ∆*asc1* cells is unaltered at the transcriptional level^45^, the observation suggested destabilization of Hel2 at the protein level. To test this, CHX-chase experiments were performed. Indeed, the rate of Hel2 turnover in ∆*asc1* cells was enhanced about two-fold when compared to the wild type (Fig. 5b, c). To test if Hel2 was degraded by the proteasome, we employed the proteasome inhibitor MG132 and ∆*erg6* background strains (termed wild type^*^ and ∆asc1^*^), because the ∆*erg6* mutation confers permeability for small molecule inhibitors such as MG132^46,47^. The analysis revealed that inhibition of the proteasome significantly increased the stability of Hel2 in wild type^*^ as well as ∆asc1^*^ cells (Fig. 5d, e). We conclude that Hel2 was turned over by the proteasome and that turnover was enhanced when Asc1 was absent, possibly because Hel2 was protected from degradation when associated with collided disomes and trisomes.

### Hel2-dependent polyubiquitination of Rps20 in vivo

It was initially demonstrated that Hel2-dependent K63-linked ubiquitination plays a crucial role in the NGD pathway^7^. Further investigations revealed that Hel2 and ZNF598 target and ubiquitinate Rps20, Rps10, and Rps3 within stalled ribosomes^16–18^. With regard to Rps20, the Inada laboratory initially demonstrated that Hel2 is responsible for mono- and di-ubiquitination of Rps20 at lysine residues K6 and K8^10^. Their preliminary findings suggested that Hel2 attaches K48-linked ubiquitin chains to Rps20 in vivo. However, subsequent research by the same group showed that Rps20 is modified with both K48- and K63-linked ubiquitin chains^16^. In contrast, reconstituted translation and ubiquitination assays revealed enrichment of K63-linked chains in ubiquitinated disomes. These K63-linked chains were found to play a crucial role in the downstream steps of the RQC pathway^14^.

**Fig. 6:**
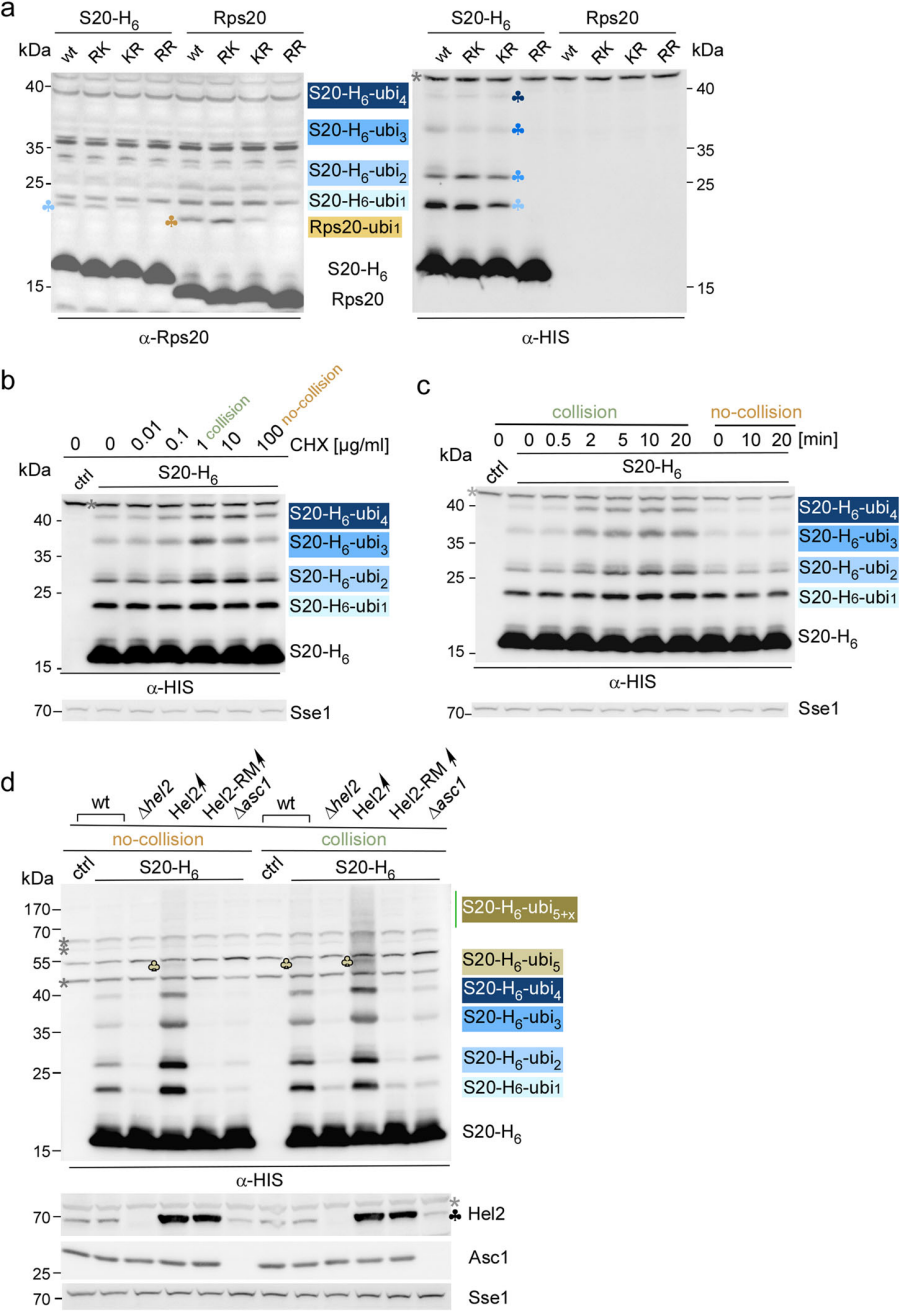
Hel2-dependent in vivo ubiquitination of Rps20. **a** A fraction of Rps20 is polyubiquitinated in unstressed log-phase cells. Lysate from wild type or S20-H_6_ cells carrying single K6R (RK), K8R (KR), or double K6R, K8R (RR) mutations. Immunoblots were analyzed with α-Rps20 (left panel, Fig. S6a) or α-HIS (right panel). The length of ubiquitin chains (ubi_1–__4_) attached to S20-H_6_ is indicated. Background bands above 40 kDa on α-HIS immunoblots are labeled with gray asterisks. **b** CHX induces polyubiquitination of S20-H_6_ in a concentration dependent manner. Log-phase S20-H_6_ cells were treated for 10 min with the indicated CHX concentration. **c** CHX-induced polyubiquitination of S20-H_6_ reaches a plateau. S20-H_6_ cells were treated with low-dose CHX (collision) or were left untreated (no-collision). **d** Polyubiquitination of Rps20 in log-phase cells depends on catalytically active Hel2 and on ribosomal protein Asc1. Lysates from log-phase cell cultures of the indicated strains in the S20-H_6_ background without treatment (no-collision) or after a 10 min treatment with 1 µg/ml CHX (collision). Untagged wild type served as a control (ctrl). For details see Methods. Immunoblots were decorated with α-HIS, α-Hel2, α-Asc1, and α-Sse1 (loading control). The length of the ubiquitin chain attached to S20-H_6_ is indicated; ubi_5+x_ indicates the smear of polyubiquitinated S20-H_6_ species attached to more than 5 ubiquitin residues.

We revisited the in vivo ubiquitination of Rps20 with an experimental setup preserving translational status and protein ubiquitination (see Methods). Initial experiments were performed with log-phase wild type and mutant strains, in which Rps20 lysine 6 (Rps20-RK), lysine 8 (Rps20-KR), or both lysine residues (Rps20-RR) were genomically replaced by arginine (Fig. 6a). An antibody directed against Rps20 (α-Rps20) revealed mono-ubiquitination of wild-type Rps20, as well as the Rps20-RK and Rps20-KR mutants. However, ubiquitination of the double mutant Rps20-RR was not detected (Fig. 6a, left panel). The level of mono-ubiquitination of the Rps20 RK and KR single mutants was similar when compared to the wild type, suggesting that Hel2 attached ubiquitin to either K6 or K8, but not to both lysine residues. Unfortunately, the detection of more highly ubiquitinated Rps20 species was hampered by strong cross-reactions above 20 kDa (Fig. 6a, left panel and Fig. S6a). Thus, a C-terminal hexahistidine-tag was genomically fused to Rps20 (S20-H_6_) and to the RK, KR, and RR versions of Rps20 (Figs. 6a and Fig. S6a, b). As α-HIS showed little background in the lower molecular mass region, it allowed for the detection of mono- to tetra-ubiquitinated S20-H_6_ species (S20-H_6_-ubi_1__–__4_) in unstressed wild type cells. The polyubiquitination pattern of the single RK and KR mutants was similar to S20-H_6_, however, S20-H_6_-RR lacked ubiquitination (Fig. 6a, right panel). To test for the linkage between ubiquitin moieties, Rps20-H_6_ was affinity purified from low-dose CHX treated cells. Immunoblotting with a general ubiquitin antibody confirmed that S20-H_6_ was covalently attached to at least one to four ubiquitin moieties (Fig. S6c, α-ubi). S20-H_6_-ubi_2__–__4_, but not S20-H_6_-ubi_1_, were detected by linkage-specific α-K63-ubi, but were not detected by α-K48-ubi (Fig. S6c, α-K63-ubi and α-K48-ubi). We conclude that the main part of ubiquitinated Rps20 was attached to a K63-linked chain consisting of up to four ubiquitin moieties. Titration with increasing concentrations of CHX revealed that polyubiquitination of Rps20 reached a maximum at 1 µg/ml CHX (Fig. 6b). This is consistent with previous studies demonstrating that intermediate concentrations of translation inhibitors saturate the RQC pathway^24^, induce mono-ubiquitination of Rps3^9^, ribosome stalling, and the general stress response^48^. When the CHX concentration was increased to 100 µg/ml, which corresponds to high-dose CHX, polyubiquitination of Rps20 resembled that of untreated cells, because no further collisions occur when all ribosomes stall (Fig. 6b). A time course with low-dose CHX revealed that the level of S20-H_6_-ubi_1__–__4_ reached a plateau after 5 min (Fig. 6c).

S20-H_6_ was introduced into the genome of ∆*hel2*, ∆*asc1*, Hel2 ↑ , and into a strain overexpressing Hel2-RM in the ∆*hel2* background (Hel2-RM ↑ ). As expected, Rps20 polyubiquitination was absent in ∆*hel2* and Hel2-RM↑ strains (Fig. 6d). This confirmed that Hel2 is essential for ubiquitination of Rps20^10^ and that the Hel2-RM mutant was indeed catalytically inactive (related to Figs. 3a, b and  4d). Polyubiquitination of Rps20-H_6_ in ∆*asc1* cells was also strongly reduced, however, was slightly higher compared to ∆*hel2* and Hel2-RM↑ cells, particularly after induction of collision by low-dose CHX (Fig. 6d, see Discussion). In the wild type, low-dose CHX treatment raised the concentration of S20-H_6_-ubi_1–__4_ and S20-H_6_-ubi_5_ and more highly polyubiquitinated species (S20-H_6_-ubi_5+x_) appeared (Fig. 6d). Considering that a single lysine residue (K6 or K8) was sufficient to attach polyubiquitin chains to Rps20 (Fig. 6a), the result demonstrates that induction of collision, increased the overall number of ubiquitinated Rps20 molecules and the length of ubiquitin chains attached to Rps20 (Fig. 6d). Rps20 polyubiquitination (Fig. 6b, d) was induced by the same CHX concentration, which also induced salt-resistant binding of Hel2 to disomes/trisomes (Fig. 2d), formation of disomes/trisomes (Fig. 4b) and Hel2 occupancy on trisomes (Fig. 4e). Notably, increased Rps20 polyubiquitination was also observed in the Hel2↑ strain (Fig. 6d), which contained wild type levels of disomes and trisomes (Fig. 1b). This is consistent with a model, in which the occupancy of disomes, which is only about 5% in a wild type background (Fig. 4e), was strongly increased due to overexpression of Hel2 (Fig. S1c).

### Rps20 is deubiquitinated upon translational run-off

**Fig. 7:**
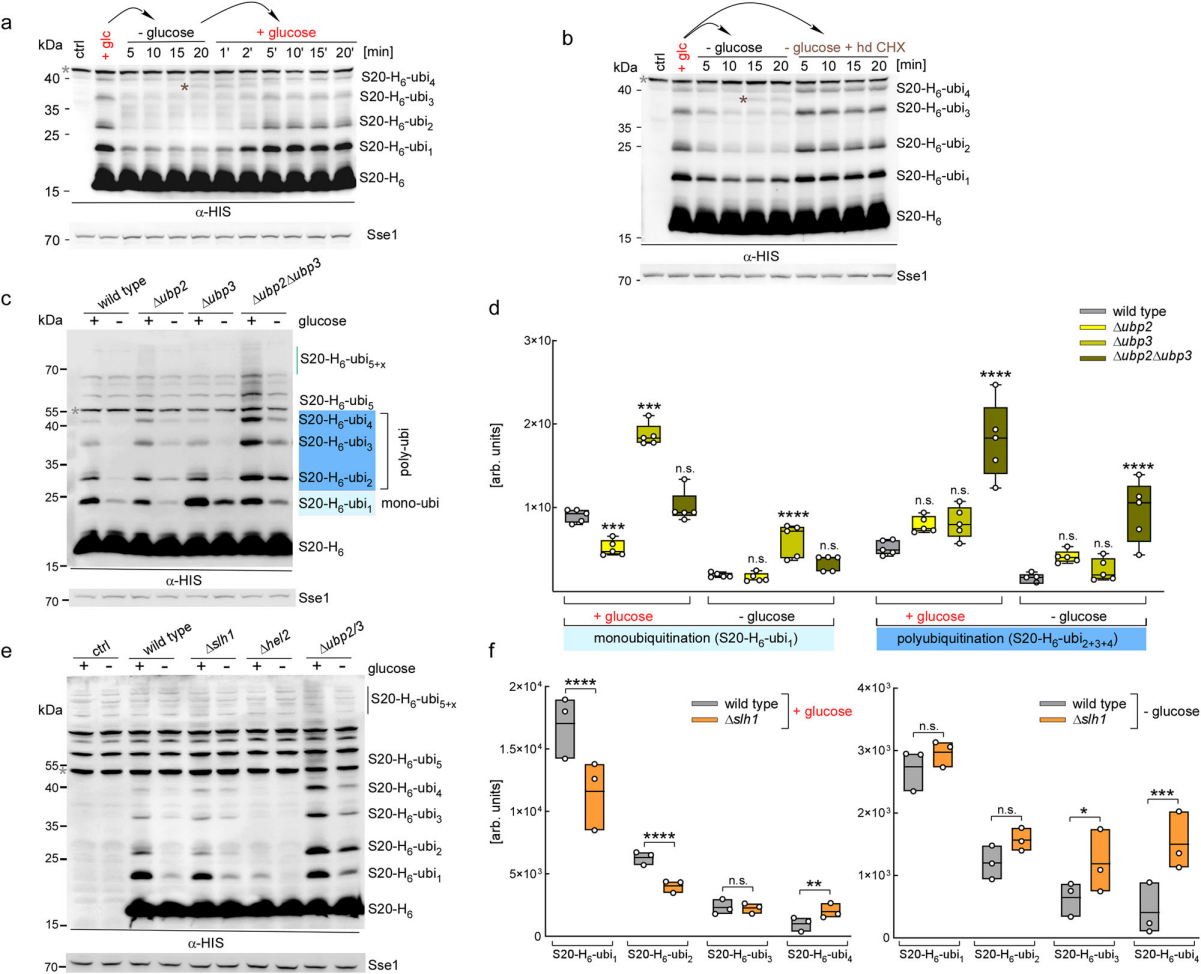
Deubiquitination of Rps20 is affected by Ubp2, Ubp3, and Slh1. **a** Rps20 is deubiquitinated upon translational run-off. Glucose-depletion/re-addition experiments were performed with S20-H_6_ strains as described in Methods. Untagged wild type served as control (ctrl). S20-H_6_ and polyubiquitinated S20-H_6_ species were analyzed in lysates by immunoblotting with α-HIS; Sse1 served as loading control. Gray asterisks indicate background bands recognized by α-HIS, brown asterisks indicate a background band, which appeared only in glucose depleted cells (Fig. S7a). **b** Deubiquitination of S20-H_6_ is blocked by high-dose CHX. S20-H_6_ cells were transferred to glucose-free medium, or to glucose-free medium supplemented with high-dose (hd) CHX as described in (**a**). **c** Ubiquitination pattern of S20-H_6_ in strains lacking Ubp2 and/or Ubp3. Log-phase cells of S20-H_6_ and ∆*ubp2*, ∆*ubp3*, ∆*ubp2*∆*ubp3* in the S20-H_6_ background were flash-frozen directly ( + glucose) or after 5 min glucose depletion (- glucose). The length of the polyubiquitin chains (ubi_1_ to ubi_5+x_) is indicated. **d** Ubp2 and Ubp3 mediate deubiquitination of Rps20. Shown is a Min to Max plot of Rps20-H_6_ mono- and polyubiquitination during log-phase and after glucose depletion. Statistical analysis was performed on the basis of 5 independent experiments as shown in **c**. S20-H_6_-ubi_1_ represents monoubiquitinated Rps20; the sum of S20-H_6_-ubi_2+3+4_ bands represent poly-ubi. Higher molecular mass polyubiquitinated S20-H_6_ species were excluded from the analysis. *p*-values represent the comparison between wild type and each mutant strain. **e** Ubiquitination pattern of S20-H_6_ in log-phase and glucose depleted ∆*slh1*, ∆*hel2*, and ∆*ubp2*∆*ubp3* cells as in **c**. **f** Slh1 is required for efficient deubiquitination of tri- and tetra-ubiquitinated Rps20 species. Shown are Min to Max plots of Rps20-H_6_-ubi_1–4_ levels during log-phase (left panel) and after glucose depletion (right panel). Statistical analysis was performed on the basis of 3 independent experiments shown in Fig. S7c. For details see Methods. Lines in boxes indicate the mean. n.s.: *p* > 0.05, * *p*: < 0.05, **: *p* < 0.01, ***: *p* < 0.001.

Wild type cells maintained a steady concentration of disomes (Figs. 1–3 and Fig. 4a) as well as a constant level of polyubiquitinated Rps20 (Figs. 6c and Fig. S6c). This suggested an equilibrium between ubiquitination and deubiquitination of Rps20. To test if the resolution of disomes/trisomes affected Rps20 ubiquitination, we performed glucose depletion (Fig. 1a, c) and then analyzed the ubiquitination pattern of Rps20. Only 5 min after glucose withdrawal, the level of Rps20 polyubiquitination was strongly reduced (Figs. 7a and  S7a). Deubiquitination of Rps20 was blocked by high-dose CHX, indicating that deubiquitination was dependent on translational run-off (Fig. 7b). Conversely, the steady-state level of S20-H_6_-ubi_1–4_ was restored 5 minutes after glucose was reintroduced to the growth medium (Fig. 7a). We conclude that Hel2-dependent polyubiquitination of Rps20 ceases when disomes and trisomes resolve.

### Rps20 deubiquitination is mediated by Ubp2 and Ubp3

Deubiquitination of Rps20 requires one or more deubiquitinating enzymes (DUBs), which cleave K63-linked polyubiquitin chains (Fig. S6c). In the mammalian system it was shown that USP21 and OTUD3 can directly antagonize ZNF598-mediated ubiquitination of Rps20/uS10 and Rps10/eS10 ^49^. However, in the yeast it is unclear which DUBs are responsible for the deubiquitination of Rps20. The yeast DUBs Ubp2 and Ubp3 hydrolyze K63-linked ubiquitin chains^50–52^. Ubp3 binds to ribosomes^53^, and Ubp2 as well as Ubp3 were detected in close proximity of Asc1^54^. Of note, Ubp3 hydrolyzes substrate-monoubiquitin bonds^50^, while Ubp2 does not^51^. We generated ∆*ubp2*, ∆*ubp3*, and ∆*ubp2*∆*ubp3* strains in the wild type and S20-H_6_ background. All deletion strains were hypersensitive to CHX. The ∆*ubp3* strain was more sensitive than the ∆*ubp2* strain, while the ∆*ubp2*∆*ubp3* double deletion strain showed most pronounced CHX sensitivity (Fig. S7b). We note that the ∆*ubp2*,∆*ubp3*, and ∆*ubp2*∆*ubp3* strains displayed more severe CHX sensitivity when compared to ∆*hel2* and Hel2-RM strains (Fig. S7b). This may indicate that the accumulation of polyubiquitinated Rps20 (Fig. 7c) is more harmful than loss of RQC, possibly because the latter can be compensated by read-through of stalling-prone sequences^4,8,12,20,21,24,25^. Alternatively, increased CHX sensitivity of strains lacking Ubp2 and/or Ubp3 could be connected to additional functions of the DUBs^50–53^.

We next tested Rps20 mono-ubiquitination (S20-H_6_-ubi_1_) and polyubiquitination (S20-H_6_-ubi_2–4_, see Methods, Fig. 7c, d) in the ∆*ubp2*, ∆*ubp3*, and ∆*ubp2*∆*ubp3* strains. Mono-ubiquitination of Rps20 was significantly increased in the ∆*ubp3* strain, in which S20-H_6_-ubi_1_ accumulated in log-phase cells, as well as after glucose depletion (Fig. 7c, d). Thus, Ubp3 played a major role in the hydrolysis of the Rps20 monoubiquitin bond^50^. In contrast, accumulation of polyubiquitinated Rps20 was most pronounced in the ∆*ubp2*∆*ubp3* double deletion strain (Fig. 7c, d), suggesting that both, Ubp2 and Ubp3 contributed to the cleavage of K63-linked polyubiquitin chains. The ∆*ubp2*∆*ubp3* strain also accumulated more highly polyubiquitinated Rps20 species, which were difficult to quantify (Fig. 7c, S20-H_6_-ubi_5+x_). We note, however, that even in the ∆*ubp2*∆*ubp3* strain glucose depletion resulted in deubiquitination of Rps20, though with reduced rate (Fig. 7c, d). Additional DUBs thus likely contribute to Rps20 deubiquitination. In any case, the combined data reveal that Ubp2 and Ubp3 are required to maintain the steady Rps20 polyubiquitination level in wild type log-phase cells and contribute to the fast deubiquitination of Rps20 upon translational run-off.

### Ubiquitin chain length on Rps20 indicates whether disome/trisome resolution depends on the RQC pathway

To test if the RQC machinery was required for Rps20 deubiquitination upon translational run-off we employed a ∆*slh1* strain (Fig. S7b). Slh1, a subunit of the trimeric RQT complex, acts downstream of Hel2 and is required for the splitting of stalled ribosomes^10,16,17,20^ (see Introduction). In log-phase, the overall pattern of Rps20 ubiquitination in wild type and the ∆*slh1* strain was similar (Figs. 7e and  S7c, d). Upon closer inspection, however, in ∆*slh1* cells the steady state level of mono- and di-ubiquitinated Rps20 species was slightly reduced, while tri- and tetra-ubiquitinated Rps20 species were increased (Figs. 7e and S7c). The finding suggested that Hel2 kept extending the ubiquitin chain on Rps20 if disomes/trisomes persisted in the absence of Slh1. Consistently, when glucose was depleted, mono- and di-ubiquitinated Rps20 species dropped to a low-level in wild type as well as in the ∆*slh1* strain (Fig. 7e, f). However, tri- and tetra-ubiquitinated Rps20 species were stabilized in the absence of Slh1 (Fig. 7e, f). Thus, deubiquitination of S20-ubi_1_ and S20-ubi_2_ was largely Slh1-independent, whereas deubiquitination of S20-H6-ubi_3_ and, to an even greater extent, S20-H6-ubi_4_ was Slh1-dependent. (Figs. 7e and  S7c, d compare S20-H_6_-ubi_3_ and S20-H_6_-ubi_4_ bands in wild type and ∆*slh1* - glucose). The data reveal that the lifespan of disomes and trisomes determines the length of the Hel2-dependent ubiquitin chain attached to Rps20. Disomes/trisomes that resume translation quickly receive only one or two ubiquitin moieties, whereas more severely stalled disomes/trisomes acquire longer ubiquitin chains. The model is consistent with the reported requirement of Slh1 for the resolution of disomes/trisomes trapped on strong stalling reporters in vivo and in vitro^10,16,17,20^. The model also provides a mechanism by which Hel2/ZNF598 may act as a timer, as previously hypothesized^17,35^. Based on the collective findings, we propose that ubiquitination of Rps20 continues as long as Hel2 remains bound to stalled disomes/trisomes. The process terminates when transiently stalled disomes resume translation or when more persistently stalled disomes are resolved by the RQC machinery. Whether the RQT complex directly recognizes the length of the polyubiquitin chain attached to Rps20 awaits further investigation.

## Discussion

In the initial stage of the RQC and NGD pathways, Hel2 identifies and polyubiquitinates RNase-resistant disomes/trisomes, which result from undesired ribosome stalling^16–18^. In this study, we characterized and quantified Hel2•ribosome complexes formed in vivo and examined the dynamics of Rps20 ubiquitination under various growth conditions. Our findings complement existing published data and provide novel insights into how Hel2 may label long-term stalled ribosome complexes for the RQC pathway.

Contrary to our initial expectation, ribosome-binding of endogenous Hel2 was not confined to RNase-resistant disomes/trisomes. In extracts prepared from log-phase cells as much as half of Hel2 was associated with translating monosomes, the other half was bound to RNase-resistant disomes and trisomes (Figs. 1a and  4a). Taking into account that translating ribosomes were approximately 13-fold more abundant than RNase-resistant disomes/trisomes (Supplementary Data 1) this indicates that the affinity_app_ of Hel2 for translating monosomes was low when compared to disomes and trisomes. As a result, Hel2 occupancy on disomes/trisomes was approximately 10-fold higher compared to translating monosomes (Fig. 4e). Hel2 was released from translating monosomes and most disomes/trisomes in the presence of high concentrations of salt when ribosomal complexes were isolated from log-phase cells (Fig. 2d). Under these conditions a small fraction of Rps20 was linked to 1–4 ubiquitin moieties, whereby mono- and di-ubiquitinated Rps20 species were significantly more abundant than tri- and tetra-ubiquitinated Rps20 species (Fig. 6a, d).

The interaction of Hel2 with disomes and trisomes, and to a lesser extend also translating monosomes was dependent on Asc1 (Fig. 3c, d). However, even in the absence of Asc1, Hel2 was associated with translating monosomes indicating that ribosome binding of Hel2 was not strictly dependent on Asc1. These observations are consistent with previous data obtained by in vivo crosslinking and analysis of cDNA (CRAC), which revealed that Asc1 is required for normal recruitment of Hel2 to ribosomes^37^. Of note, we found that Hel2 did not bind to non-translating monosomes (Fig. 1a, c) suggesting that the interaction with translating monosomes was mediated by interaction of Hel2 with translated mRNA. This possibility is consistent with the observation that Hel2 crosslinks to mRNA and the level of crosslinking correlates with ribosome occupancy, indicating that Hel2 contacts translated mRNA without preference for stalling-prone sequences^37^. Moreover, Hel2 forms multiple crosslinks to 18S rRNA some of which are inaccessible in disome structures, suggesting that these crosslinks originate from Hel2 associated with a translating monosome^37^.

Polyubiquitination of Rps20 in ∆*asc1* cells was significantly reduced (Fig. 6d) but not entirely abolished (Fig. 6d). This observation aligns with the finding that Hel2 was not enriched on ∆*asc1*-disomes/trisomes (Figs. 3c and  4c). However, Asc1 was not necessary for the formation of RNase-resistant disomes and trisomes. Instead, the concentration of disomes and trisomes in ∆*asc1* cells was significantly higher compared to the wild type (Fig. 4a, c). The combined data clarify seemingly contradictory observations regarding Asc1’s role in the RQC pathway. On one hand, it was suggested that Asc1 is essential for the formation of collided disomes^16,18^. Consistent with this notion, multiple studies demonstrated that read-through of stalling sequences occurs more frequently in ∆*asc1* cells compared to the wild type^55^. On the other hand, ribosome stalling persists in the absence of Asc1^2,20^. Based on our findings, we propose that Asc1 is not required for the formation of collided disomes/trisomes; however, is necessary to provide the Asc1-Asc1 platform, which enables high-affinity binding of Hel2. In the absence of the Asc1-Asc1 platform, Hel2 was primarily bound to translating monosomes (Figs. 3c and 4c), which were present in large excess over disomes/trisomes (Fig. 4e), and consequently was unavailable for the ubiquitination of stalled ribosomes (Fig. 6d).

Conditions that induced ribosome stalling or blocked the RQC pathway had little effect on the cellular concentration of disomes; however, the concentration of trisomes increased substantially (Fig. 4a–d). A plausible explanation for this observation is that, although more ribosomes stall for prolonged periods of time, and stable disomes form, these quickly convert into trisomes due to collisions with trailing ribosomes. Assuming that long-term stalling promotes the formation of the Asc1-Asc1 platform in disomes and trisomes (see Introduction), the above suggests that a higher proportion of trisomes, compared to disomes, possesses the Asc1-Asc1 platform. This would explain why the Hel2 occupancy on trisomes was extraordinarily high (Fig. 7e). An alternative explanation for the high affinity_app_ of Hel2 for trisomes, compared to disomes, is that trisomes possess additional structural features important for high affinity Hel2 binding. It should be noted that the distribution of Hel2 among different ribosomal complexes has not been previously assessed in a similar manner. However, our findings are consistent with in vitro studies, which revealed that trisomes are more efficiently polyubiquitinated by Hel2 compared to disomes^14,17^.

**Fig. 8:**
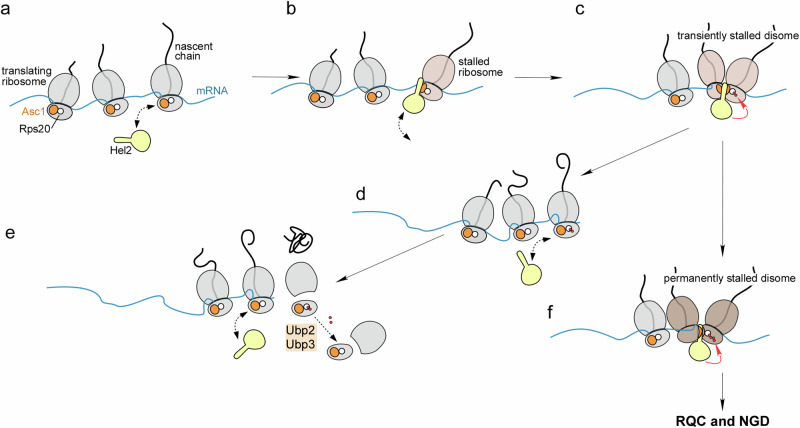
Model of dwell time dependent selection of disomes/trisomes for the RQC pathway. **a** Hel2 binds to translating ribosomes with low affinity. **b** A translating ribosome encounters a stalling-prone mRNA sequence. **c** A trailing ribosome catches up and collides with the stalled ribosome forming a disome. Hel2 binds with increased affinity to the disome and initiates ubiquitination of the 40S ribosomal protein Rps20. **d** The leading ribosome resumes translation, Hel2 reverts to its low-affinity binding mode, and ubiquitination of Rps20 ceases. **e** The deubiquitinases Ubp2 and Ubp3 remove ubiquitin chains from Rps20. This may occur during translation and/or after translation termination. **f** In case the leading ribosome stalls for a prolonged period of time, the disome develops the Asc1-Asc1 platform, which results in further increased affinity of Hel2. Hel2 now continuously extends the polyubiquitin chain until the RQC and NGD machineries are recruited and resolve the stalled ribosome. Hel2 in yellow, Asc1 in orange, Rps20 in white, ubiquitin moieties in red. For details see Discussion.

Based on our findings and published evidence, we propose that Hel2 monitors the persistence of stalled ribosomes (Fig. 8). Hel2 binds to translating ribosomes with low affinity. In this mode, ribosome binding is mediated mainly by interactions with rRNA, mRNA, and possibly the Asc1-region of the 40S ribosomal subunit^37^ (Fig. 8a). Hel2 binds likely to monosomes with high on/off rate, allowing it to scan for ribosome stalling until a trailing ribosome collides with a stalled leading ribosome (Fig. 8b). Collisions lead to the formation of disomes with a specific 40S–40S interface, which may initially lack a fully established Asc1-Asc1 platform^28^. Hel2 now binds with increased affinity/stability and ubiquitinates Rps20 (Fig. 8c). Once the leading ribosome resumes translation, ubiquitination of Rps20 ceases, and Hel2 returns to the low-affinity binding mode (Fig. 8d). The action of Ubp2/Ubp3 now prevails and ubiquitin chains are removed from Rps20. Deubiquitination may occur continuously, or shortly after translation termination (Fig. 8e). When a leading ribosome stalls for an extended period of time the Asc1-Asc1 platform between collided ribosomes forms. Hel2 then binds with high affinity and in a salt-resistant manner (Fig. 8f). Formation of the Asc1-Asc1 platform might be a continuous process, that also depends on the cause of ribosome stalling^3,13,14,28,36^. Extended dwell times of disomes and trisomes enable Hel2 to continuously extend the ubiquitin chain on Rps20, which ultimately recruits the RQC/NGD machineries and leads to the disposal of stalled ribosomes (Fig. 8f).

## Methods

### Plasmids

For the construction of pYCplac111-HEL2 and pYEplac181-HEL2 *HEL2* plus 486 bp up- and 206 bp down-stream was cloned into the HindIII/PstI sites of pYCplac111 or pYEplac181, respectively. QuickChange mutagenesis (Stratagene) was used to generate the yeast expression plasmids pYCplac111-Hel2-C64S and pYEplac181-Hel2-C64S by exchange of guanine in position 189 of *HEL2* to cytosine. Hel2-C64S is catalytically inactive and is termed Hel2-RM (Fig. 6d). Plasmids are listed in Table S1.

### Yeast strains

MH272–3 fα (*ura3*, *leu2*, *his3*, *trp1*, *ade2*)^56^ is the wild type background for all mutant strains employed in this study. Gene deletion and epitope-tagging was performed by homologous recombination^57,58^. Proper integration of deletion and tagging cassettes into the genome was verified by PCR/sequencing and immunoblotting with antibodies directed against the translation product of the respective gene (Figs. S1b and  6a). Yeast strains are listed in Table S2.

### Generation of Hel2-RM_g_

For the generation of genomically integrated catalytically inactive Hel2-C64S, we made use of the observation, that read-through of stalling-prone sequences, such as for example poly-arginine encoding (*CGA*)_12_ sequences, is enhanced in the absence of active Hel2^25^. The wild type MH272-3fα strain cannot grow on minimal media lacking leucine, because of the *leu2* mutation (Table S2). The plasmid pYEplac195-Luc-R12_(CGA)_-Leu2 encodes for a fusion between firefly luciferase (Luc) and beta-isopropylmalate dehydrogenase, the gene product of *LEU2*. Luc and Leu2 are separated by the stalling-prone (*CGA*)_12_ sequence pYEplac195-Luc-R12_(CGA)_-Leu2 does not support growth of the MH272-3fα wild type on minimal medium lacking leucine, as too little of the full-length fusion protein is made. To generate Hel2-C64S, MH272-3fα was co-transformed with pYEplac195-Luc-R12_(CGA)_-Leu2 and the integrable PCR product HEL2-C64S (Table S3). Transformants were selected on SD medium supplemented with 10 µg/ml leucine (low leucine), instead of the routinely used 100 µg/ml leucine. Transformants growing on low leucine were selected and the genomic Hel2-C64S point mutation was confirmed by sequencing. A clone, which had lost pYEplac195-Luc-R12_(CGA)_-Leu2, and was now unable to grow in the absence of uracil, was termed Hel2-RM_g_ (Table S3), and was used for further experiments.

### Generation of S20-H_6_ strains

Rps20 was C-terminally tagged by integration of the 6x*HIS-KanMX4* cassette, which was generated by PCR using plasmid pYM46^58^ as template (Table S1). A Ser-Ser-Gly linker was introduced between the most C-terminal residue of Rps20 (Asn121) and the His_6_ epitope. Tagging of Rps20 was essential for the detection of polyubiquitinated Rps20 species (Figs. 6a and S6a).

### Generation of Rps20-K6R (RK), -K8R (KR), and -K6R-K8R (RR)

pRCC-N-Rps20 PAM23 (Table S1) was generated by Gibson assembly^59^. Rps20 point mutations K6R and K8R were generated by CRISPR-Cas genome editing^59^. Protospacers (20 bases) were chosen according to the CRISPR gRNA Design tool provided by ATUM and were cloned into the CRISPR-Cas9 vector pRCC-N^59^ (Table S1). The resulting pRCC-N-Rps20 PAM23 plasmids were cotransformed with the respective donor DNA (Table S3) into wild type or S20-H_6_ cells. Genomic point mutations were confirmed by PCR-amplification of the genomic DNA region followed by sequencing (Eurofins).

### Media and general growth conditions

Yeast cells were grown at 30 °C in YPD medium (1% yeast extract, 2% peptone, 2% glucose, 20 mg/ml adenine-hemisulfate) at 200 rpm on a shaker and were harvested in log-phase. G418 (final concentration 300 µg/ml) or nourseothricin (final concentration 100 µg/ml) was added to YPD medium as required. Glucose depletion was performed in YP medium (1% yeast extract, 2% peptone, 20 mg/ml adenine-hemisulfate) at 200 rpm on a shaker for the times indicated. Pre-cultures and cultures for experiments involving MG132-treatment were grown on SD minimal medium (6.7 g/l yeast nitrogen base w/o amino acids, 2% glucose, supplements as required).

### No-collision treatment and harvest of yeast cultures

Aim of no-collision treatment was to preserve the in vivo translational status of log-phase ribosomes as closely as possible. Particularly, to maintain the in vivo collided disome concentration and to prevent undesired translational run-off. For this purpose, we employed two methods, depending on the cell culture volume and the cell disruption method. The first method was used for the preparation of yeast extract (see below), which requires culture volumes of 50–100 ml and releases cellular proteins in the native state. The second method was used for the preparation of yeast lysate (see below), which requires a culture volume of 1–1.5 ml and denatures cellular proteins directly upon cell lysis. To prepare no-collision yeast extract, high-dose CHX (final concentration of 100 µg/ml) was added to log-phase cultures at 30°C, which were then incubated for 5 min on ice and subsequently collected at 4°C and 4500 × *g* for 1 min. This treatment blocks translation elongation^60^, prevents translational run-off ^38^, and preserves pre-existing collided disomes during yeast extract preparation. For the preparation of no-collision yeast lysate 10 ml yeast cultures were grown to log-phase and 1–1.5 ml of these cultures was collected by centrifugation at 4°C and 20,000 × *g* for 15 s. The supernatant was removed, cell pellets were flash-frozen in liquid nitrogen, and were stored at −80 °C. Please note, for the preparation of yeast lysate, treatment with high-dose CHX was omitted, because rapid cell harvest, followed by flash-freezing and subsequent protein denaturation prevents translational run-off.

### Induction of collision or translational run-off prior to harvest of yeast cultures

To induce ribosome stalling, low-dose CHX (final concentration 0.5–1 µg/ml)^9,24^ was added to log-phase cultures on a shaker at 200 rpm and 30°C. Incubation with low-dose CHX was continued for 10 min, if not indicated otherwise. The treatment was used for subsequent preparation of yeast extract as well as yeast lysate. Translational run-off was induced by glucose depletion^38^. For the preparation of glucose-depleted yeast extract cells from 50 ml cultures were collected at room temperature, were washed with 1 culture-volume YP (pre-warmed to 30 °C), and were then resuspended in 1 culture-volume pre-warmed YP. After 10 min incubation on a shaker at 30 °C, high-dose CHX was added and cells were collected as described above. For the preparation of glucose-depleted yeast lysate 1–1.5 ml of log-phase cultures were collected by centrifugation at 20 °C and 20,000 × *g* for 15 s, cell pellets were resuspended in 1 ml pre-warmed YP, and were incubated at 30 °C in a Thermomixer (Eppendorf) at 1400 rpm for 3 min. Cells were then re-collected by a 15 s spin and were shock-frozen in liquid nitrogen. The total time for this procedure was 5 min, which corresponds to 5 min glucose depletion, because glucose depletion conditions are established already during cell harvest. Different glucose depletion times were achieved by adjusting the incubation time in YP medium. Glucose re-addition experiments were performed by adding glucose directly to glucose-depleted cell cultures to a final concentration of 2% glucose.

### Preparation of yeast extract and RNase treatment

Yeast extract was used for sucrose gradient sedimentation and ribosome binding experiments. The extract was generated by the glass beads method as previously described^38,61^. If not indicated otherwise log-phase cultures were treated with high-dose CHX as described above. Subsequently, cells ( ~ 50 OD_600_ units) were collected by centrifugation, cell pellets were washed with 20 ml ribosome-binding buffer (40 mM HEPES-KOH pH 7.4, 120 mM KAc, 2 mM MgAc_2_, 2 mM dithiothreitol (DTT), 50 µg/ml CHX, 1 mM PMSF and protease inhibitor mix (1.25 μg ml^−1^ leupeptin, 0.75 μg ml^−1^ antipain, 0.25 μg ml^−1^ chymostatin, 0.25 μg ml^−1^ elastinal, 5 μg ml^−1^ pepstatin A), and were then resuspended in 600 µl ribosome-binding buffer. Glass beads were added and disruption was performed with a Disruptor Genie (Scientific Industries) at 4 °C for 3×2-min cycles, interrupted by 2-min incubation on ice. Cell debris were removed by two 10–20 min rounds of centrifugation at 20,000 × *g* and the resulting supernatant (A_260_ ~ 70–90 mAU), which is termed yeast extract, was used for further analysis directly. RNase treatment of yeast extract was performed with RNase A (final concentration 250 µg/ml) for 10 min on ice^42,62^.

### Preparation of yeast lysate

Yeast lysate was employed for the analysis of Hel2 and polyubiquitinated S20-H_6_ expression levels. The lysate was prepared from cells collected as described above by the method of Yaffe and Schatz^63^. Frozen cell pellets corresponding to an OD_600_ of 1–1.5 were thawed on ice, resuspended in 1 ml of ice-cold H_2_O, mixed with 150 µl ice-cold, freshly prepared alkaline lysis buffer I (1.85 M NaOH, 7.5% (v/v) β-mercaptoethanol), and were then incubated for 10 min on ice. Subsequently, 150 µl 50% (w/v) trichloroacetic acid (TCA) solution was added and the mixture was incubated on ice for 10 min and then at 65°C for 5 min. TCA pellets were collected at 4°C and 20,000 × *g* for 10 min. TCA pellets were dissolved in SDS sample buffer (OD_600_ of 1 per 150 µl) in a Thermomixer at 37°C. As required, the pH of the sample was adjusted with NH_3_ gas phase. Samples were incubated at 95°C for 5 min and were cleared by centrifugation prior to analysis. Typically, aliquots corresponding to an OD_600_ of 0.2 were separated on Tris-Tricine gels^64^ followed by transfer to nitrocellulose or PVDF membrane and immunodetection.

### Sucrose gradient sedimentation

Polysome profiles were generated as previously described^38,61^. For this purpose, log-phase cells (typically from 50 ml cultures) were collected, and yeast extract (with a final volume of approximately 600 µl) was prepared as described above. Extract corresponding to 10 A_260_ units was loaded onto a 12 ml 15–55% linear (Figs. 1–3). For the quantification of monosomes/disomes/trisomes extract corresponding to 5 A_260_ units was loaded onto a 12 ml 15–40% (Fig. 4) sucrose gradient prepared as above. Gradients were centrifuged at 4 °C and 200,000 × *g* (TH641, Sorvall) for 2.5 h which were subsequently fractionated into 560 μl aliquots with a gradient fractionator monitoring A_260_ (Piston Gradient Fractionator, Biocomp). Overlaid A_260_ traces across the gradient in Figs. 1–3 were normalized to the 80S monosome peak as previously described^18^. The input corresponding to 5% of the material loaded onto the gradient and aliquots of fractions 1–20 (Figs. 1–3) or 1–14 (Fig. 4) were TCA precipitated and were analyzed by immunoblotting. We note, that the ratio between different ribosomal complexes in repeats with the same strain under the same conditions (Fig. S1a) was highly reproducible. We discuss relative affinities_app_ of Hel2 for ribosomal complexes based on comigration in polysome profiles (Figs. 1–4). We note that sucrose gradient sedimentation and ribosome-binding assays (see below) were performed under non-equilibrium conditions and that on/off rates affected the observed relative affinities_app_^65^. For example, the observation that Hel2 smears into cytosolic fractions of the sucrose gradient, when Hel2 is in excess compared to disomes, (Fig. 1b, c) or when Asc1 is absent (Fig. 3c) is compatible with the idea that Hel2 binds less stably to translating ribosomes when compared to disomes. Likewise, salt-resistant binding of Hel2 to disomes might come along with reduced on/off rates when compared to salt-sensitive binding.

### Ribosome-binding assay

Cell cultures (50 ml) were pre-treated with high-dose CHX (no-collision) or with low-dose (collision) and subsequently yeast extract was generated as described above. Ribosomes were separated from the cytosol using a sucrose cushion (25% sucrose, 40 mM HEPES-KOH, 2 mM Mg(Ac)_2_, 2 mM DTT, 1 mM PMSF, and 1× protease inhibitor mix) containing either 120 mM KAc (low salt cushion) or 800 mM KAc (high salt cushion). For that purpose, yeast extract (60 µl) was loaded onto 90 µl of low salt or high salt cushions and ribosomes were collected by ultra-centrifugation at 400,000 × *g* (TLA-100, Beckman) and 4°C for 35 min. The supernatant was collected and the ribosomal pellet was resuspended in 300 µl ribosome-binding buffer. Aliquots of the extract, the supernatant, and resuspended ribosomes were TCA precipitated and analyzed by immunoblotting.

### CHX-chase experiments

CHX-chase experiments were performed as described^66^ with minor modification. Sixty ml yeast cultures were grown to log-phase in YPD at 30°C when chase experiments were performed in the wild type background. For experiments in the ∆*erg6* background 60 ml cultures were grown at 30°C on minimal SD medium, because inhibition of the proteasome by MG132 (Sigma-Aldrich, M8699) was more effective on minimal medium. For chase experiments yeast cultures were grown in YPD medium in a shaking water bath at 30 °C and 260 rpm. CHX was added to a final concentration of 100 µg/ml to stop translation and initiate the chase. Directly after addition of CHX (t = 0 min) 1.5 ml samples were collected by centrifugation at 4°C and 20,000 × *g* for 15 s. Cell pellets were shock frozen in liquid nitrogen and stored at −80 °C. Additional samples were taken at the indicated time points. For chase experiments in the presence or absence of MG132 each 25 ml of SD cultures were transferred to 100 ml Erlenmeyer flasks in a 30 °C shaking water bath (260 rpm). Flask 1 contained 80 µl DMSO (ctrl), flask 2 contained 80 µl 25 mM MG132 in DMSO to inhibit proteasome activity (final concentration 80 µM MG132). After 30 min incubation in the shaking water bath, CHX was added to a final concentration of 100 µg/ml to inhibit translation and initiate the chase. Directly after addition of CHX (t = 0 min) 3 ml samples were collected by centrifugation at 4 °C and 20,000 × *g* for 15 s. Cell pellets were shock-frozen in liquid nitrogen and stored at −80 °C. Additional samples were taken at the time points indicated. Yeast lysate was prepared from the samples as described above, with the modification that TCA pellets from SD-grown cells were dissolved in 100 µl sample buffer per 1 OD_600_ unit. Please note that Hel2 was turned over more slowly in the ∆*erg6* background when compared to the wild type (Fig. 5d, e). Also, the Hel2 stability was less severely affected by Asc1 (Fig. 5d, e). The reason for the difference is unclear. It might be connected directly to the ∆*erg6* mutation, or to the growth conditions. CHX-chase experiments in the wild type background were performed on YPD medium, while in the ∆*erg6* background experiments had to be performed in SD minimal medium to reach effective inhibition of the proteasome.

### Denaturing Ni-NTA purification of S20-H_6_

Denaturing Ni-NTA purification was employed to determine the ubiquitin linkage within polyubiquitinated S20-H_6_^67^. To increase polyubiquitination of S20-H_6_, collision in the S20-H_6_ strain was induced with 1 µg/ml CHX at 30°C for 20 min. Cells corresponding to 50 OD_600_ units were collected and cell pellets were frozen in liquid nitrogen and stored at −80°C. After thawing, pellets were resuspended in a final volume of 5 ml ice-cold, freshly prepared alkaline lysis buffer II (0.44 M NaOH, 1.8% (v/v) β-mercaptoethanol). The suspension was incubated for 15 min on ice, denatured proteins were precipitated with 8% (w/v) TCA, pellets were collected, and dissolved in 500 µl dissociation buffer I (50 mM Tris-HCl, pH 7.5, 1% SDS, 100 µg/ml BSA, 5 mM N-ethylmaleimide, 1 mM PMSF, 1x protease inhibitor mix, bromphenolblue) on a shaker at 37°C, adjusting the pH as required with NH_3_ gas phase. Dissolved pellets (termed denatured lysate) were diluted 1:10 with denaturing IP Buffer (50 mM Tris-HCl, pH 7.5, 100 mM NaCl, 0.5% (v/v) Triton X-100, 10 mM imidazol, 5 mM N-ethylmaleimide, 1 mM PMSF, 1x protease inhibitor mix) followed by a clearing spin a 20,000 × *g* for 15 min at 4 °C. Each 1.2 ml of the cleared denatured lysate was transferred to 1.5 ml tubes containing 40 µl 1:2 Ni-NTA agarose slurry (Ni-NTA super-flow, Qiagen ID 30410). Reactions were incubated for 2 h at 4 °C on a rotating wheel, Ni-NTA beads were collected, washed once with washing buffer (50 mM Tris-HCl pH 7.5, 100 mM NaCl, 0.5% (v/v) Triton X-100, 20 mM imidazol), and were incubated at 95 °C in 45 µl 2x SDS sample buffer containing 20 mM EDTA, pH 8.0. The input represents 5% of the denatured lysate used for each Ni-NTA reaction.

### Final sample preparation, immunoblotting, and marker proteins

TCA precipitation was performed by addition of 50% (w/v) TCA to a final concentration of 5%. After centrifugation, TCA pellets were dissolved in SDS sample buffer (60 mM Tris-HCl pH 6.8, 2% SDS, 0.5 mM EDTA, 0.001% bromphenolblue, 10% glycerol, 5% (v/v) β-mercaptoethanol) and were analyzed on 10% Tris-Tricine gels, followed by immunoblotting. Sse1, Pgk1, Ubp6, and Stm1 served as loading controls. Pgk1 was used as a cytosolic marker in ribosome-binding experiments. Rps9 and Rpl4 served as ribosomal marker proteins in ribosome-binding experiments and polysome profiles.

### Immunoblotting and antibodies

Proteins were separated on 10% or 16% Tris-Tricine gels^64^ and were subsequently transferred to nitrocellulose or PVDF membrane. Immunoblots were developed by enhanced chemiluminescence^32^. Antibodies: α-Hel2 (1:2000 (Fig. S1b), α-Rps20 (1:2000) (Fig. 5a), α-Ubp6 (1:10,000) (Fig. S1b), α-Stm1 (1:10,000) (Fig. S1b), α-Asc1 (1:2000) (Fig. S1b)^68^, α-Rpl4 (1:10,000)^69^, α-Rps9 (1:5000)^32^, α-Sse1 (1:5000)^32^, α-Pgk1 (1:10,000)^70^ are rabbit polyclonal antibodies (Eurogentec, Rospert lab collection). mouse α-HIS (1:1000, BioRad, clone AD1.1.10, MCA1396), α-ubiquitin P4D1 (1:1000, Santa Cruz Biotechnology, sc-8017), α-K63-Ub clone Apu3 (1:1000, Sigma-Aldrich, 05-1308), α-K48-Ub D9D5 (1:1000, Cell Signaling Technology, 8081), mouse α-FLAG clone M2 (1:1000, Sigma, F1804), α-HA (1:2000, Santa Cruz, Y-11, sc-805), α-Luciferase (1:100,000, Sigma L0159), α-rabbit HRP (1:10,000, Pierce, 61-6520) and α-mouse HRP (1:10,000, Santa Cruz, sc-2748). Immunoblots were developed using an ECL camera (Fusion Pulse 6, Vilber).

### Statistics and reproducibility

Each experiment was performed at least twice. Statistic analysis was performed with at least three independent measures (for details see Figures). Quantitative analysis of immunoblots was with Image J 1.54 g (National Institutes of Health, USA). Hel2 distribution profiles in sucrose gradient sedimentation experiments (Figs. 1–3) were generated with sets of immunoblots processed and developed simultaneously to allow for comparison between gradient fractions 1-10 (blot 1) and 11–20 (blot 2). Hel2 bands were quantified with Image J and the sum of band intensities in fractions 1–20 was set to 100%. The intensity of Hel2 bands in each fraction was calculated as a percentage of the total. Immunoblots of repeated measures for ribosome-binding assays (Figs. 2 and 3), CHX-chase experiments (Fig. 5), and quantitative analysis of mono- and polyubiquitinated S20-H_6_ species (Fig. 7) were generated under standardized conditions; samples of each repeat were analyzed on a single immunoblot. The sum of S20-H_6-ubi2_, S20-H_6-ubi3_, S20-H_6-ubi4_ band intensities (Image J 1.54 g) was defined as polyubiquitinated S20-H_6_ (Fig. 7d). One-phase decay was used to fit the data of Hel2 chase experiments (Fig. 5b–e). Statistical analysis was performed with GraphPad Prism 10.1.1. Analysis of Variance (ANOVA) with Tukey´s test for multiple comparison was used to determine statistical significance. Statistical analysis for ribosome-binding assays was by One-way ANOVA and for analysis of S20-H_6_ mono- and polyubiquitination by One-way (Fig. 7d) or Two-way (Fig. 7f) ANOVA, respectively. Summary *p* values are defined as follows: n.s.: *p* > 0.05, *: *p* ≤ 0.05, **: *p* ≤ 0.01, ***: *p* ≤ 0.001, ****: *p* ≤ 0.0001. The quantification of ribosomal particles in Fig. 4 is based on the assumption that the A_260_ traces of RNase-treated extracts consist of discrete peaks, each following a normal distribution^27^. Peak deconvolution for quantification was manually performed as follows: (1) Individual 60S peaks were isolated by mirroring the ascending 60S branches of the A_260_ profiles. (2) Individual trisome peaks were isolated by mirroring the A_260_ values of the descending trisome branches of A_260_ profiles. (3) Individual disome peaks were isolated by subtraction of mirrored trisome A_260_ values, followed by mirroring of the isolated descending disomes branches. (4) Individual 80S peaks were isolated from 60S peaks, by subtraction of mirrored 60S A_260_ values from A_260_ profiles, followed by subtraction of mirrored descending disome branches generated in step 3. To determine the fraction of ribosomes in individual monosome, disomes, and trisome peaks, the total area under the original polysome profile (fractions 4-14) was quantified using ImageJ and set as 100%. 80S, disome, and trisome peaks are expressed as percentages of this total. Assuming a yeast cell contains 300,000 ribosomes^32^, monosomes per cell were determined by multiplying the percentage of ribosomes in individual 80S peaks by 300,000. For quantification of disomes and trisomes, the percentage of ribosomes in individual disome/trisome peaks was multiplied by 300,000 and was then divided by 2 and 3, respectively, to account for the number of ribosomes per disome/trisome unit. Hel2 distribution across ribosomal fractions was quantified using ImageJ. Band intensities of Hel2 were measured in fractions 1–14, with their sum set as 100%. The percentage of Hel2 associated with monosomes (fractions 7–9), disomes (fractions 10-12), and trisomes (fractions 13 and 14) is given. Ribosome occupancy with Hel2 was calculated assuming 3000 Hel2 molecules per cell in wild type^34^ and Hel2-RM strains, and 1500 Hel2 molecules per cell in the ∆*asc1* strain (Fig. 5a). For calculation also refer to Supplementary Data 1.

### Miscellaneous

Ni-NTA and immunopurification reactions were performed in low protein-binding tubes (Sarstedt, 72.706.600). CHX and NEM stock solutions were prepared in EtOH. MG132 stock solution was prepared in DMSO.

## Supplementary information

Supplementary Information

Description of Additional Supplementary Files

Supplementary Data 1

Supplementary Data 2

nr-reporting-summary

## Data Availability

All data supporting the findings of this study are available within the paper, Supplementary Information, and Supplementary Data. The original uncropped and unedited images of immunoblots are included in the Supplementary Information pdf. Immunoblots for statistical analysis are included as Supplementary Data 2. Biological materials (strains, plasmids, antibodies) are available from the corresponding author on reasonable request. Correspondence and requests should be addressed to sabine.rospert@biochemie.uni-freiburg.de.
