# Supplementary material for: Stalled disomes marked by Hel2-dependent ubiquitin chains undergo Ubp2/Ubp3-mediated deubiquitination upon translational run-off
Source: Commun Biol. 2025 Jan 28;8:132. doi: 10.1038/s42003-025-07569-z (PMC11775340; doi:10.1038/s42003-025-07569-z)
Supplement: Supplementary file 1 — Supplementary Information [file 42003_2025_7569_MOESM1_ESM.pdf]

SUPPLEMENTARY INFORMATION

Stalled disomes marked by Hel2-dependent ubiquitin chains undergo Ubp2/Ubp3-mediated deubiquitination upon translational run-off

Mario Scazzari[1], Ying Zhang[1], Anna Moddemann[1], and Sabine Rospert[1,2]

[1]Institute of Biochemistry and Molecular Biology, ZBMZ, Faculty of Medicine, University of Freiburg, D-79104 Freiburg, Germany; [2]BIOSS Centre for Biological Signalling Studies, and CIBSS Centre for Integrative Biological Signalling Studies, University of Freiburg, D-79104 Freiburg, Germany

SUPPLEMENTARY FIGURES

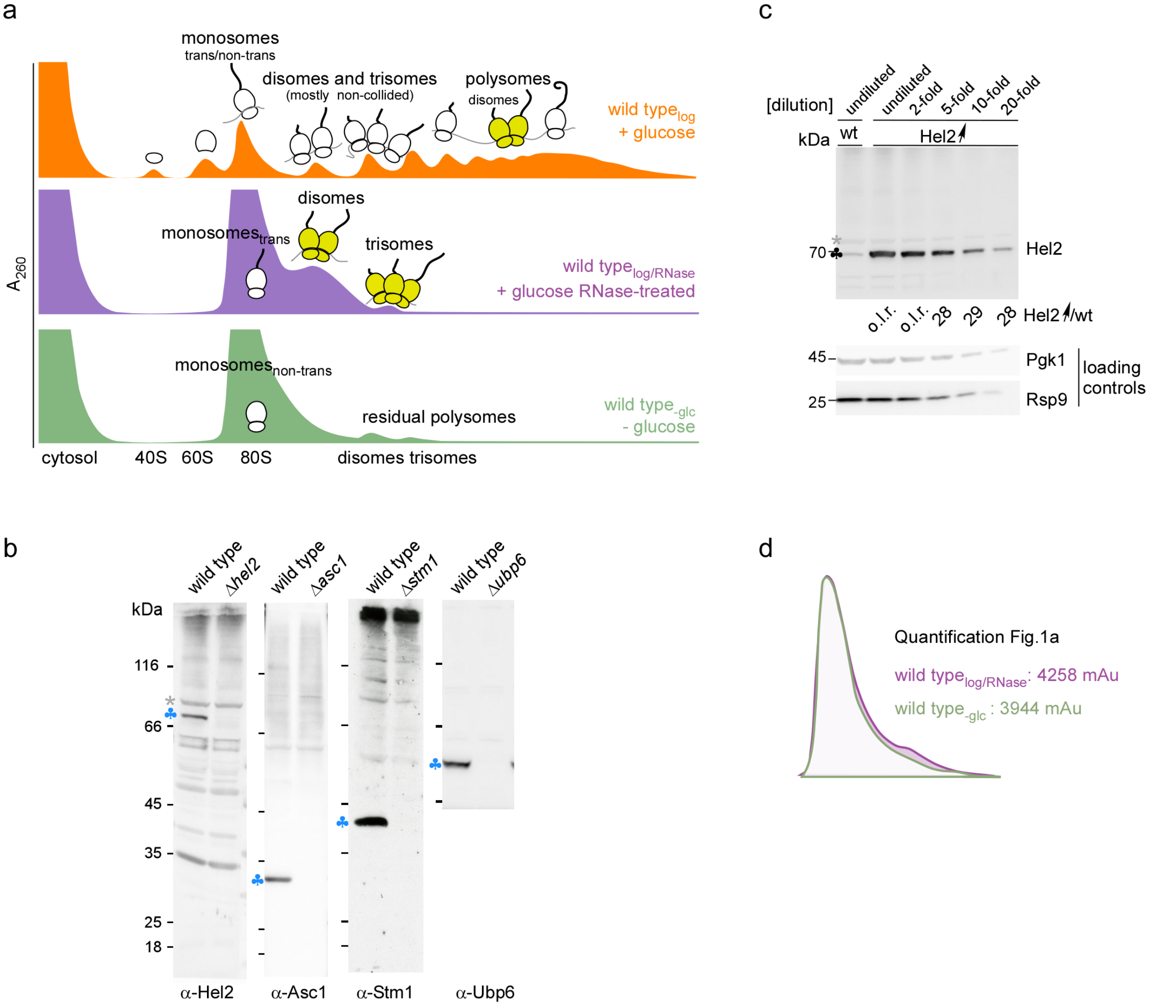

Figure S1. **Analysis of ribosomal complexes.** (**a**) Sucrose gradient sedimentation of *in-vivo* formed ribosomal complexes. **Orange model profile:** Distribution of ribosomal complexes in extract from glucose-grown log-phase cells (wild $type_{log}$). Multiple translating ribosomes scattered over a single mRNA molecule form a polysome. Disome and trisome peaks represent minimal polysomes, mostly consisting of two to three non-collided ribosomes, which are RNase-sensitive (termed disome $_{non\text{-}coll}$). The 80S peak is a mixture of translating and non-translating ribosomes, the 60S peak represents large ribosomal subunits, and the 40S peak small ribosomal subunits. **Magenta model profile:** Distribution of ribosomal complexes in RNase-treated wild $type_{log}$ extract (wild $type_{log/RNase}$). RNase digests mRNA segments connecting polysomal ribosomes and turns them into translating monosomes, which cover short mRNA segments and remain associated with peptidyl-tRNA. The mRNA connecting collided disomes/$trisomes_{coll}$ is protected from digestion [1-7]. Polysome profiles of wild $type_{log/RNase}$ lack polysomes, display a large 80S peak, consisting mainly of translating monosomes, and minor peaks consisting of RNase-resistant disomes, which overlap with the $monosome_{trans}$ peak. **Green model profile**: Distribution of ribosomal complexes in extract from glucose-depleted wild type cells (wild $type_{-glc}$). As glucose depletion causes translational run-off, wild $type_{-glc}$ extract contains few residual polysomes and a large 80S peak representing mostly $monosomes_{non\text{-}trans}$, which lack a peptidyl-tRNA [8-12]. **(b)** Antibody control blots. Lysates derived from wild type, Δ*hel2*, Δ*asc1,* Δ*stm1,* and Δ*ubp6* strains were analyzed by immunoblotting with the indicated antibodies. Bands corresponding to proteins of interest are labeled with a blue cross. The prominent background band above Hel2 is indicated by a gray asterisk throughout the manuscript. (**c**) Hel2 expression level in the Hel2↑ strain. In order to determine the expression level of Hel2 in Hel2↑ cells relative to wild type, wild type and a dilution series of Hel2↑ lysate was analyzed side by side with α-Hel2, α-Rps9, and α-Pgk1. Band intensities were determined densitometrically and were extrapolated to the undiluted Hel2↑ lysate. Numbers below the immunoblot indicate the ratio of Hel2 band intensity in undiluted Hel2↑/undiluted wild type lysate. Samples in which the Hel2 band was outside of the linear range (o.l.r.) were excluded from the calculation. **(d)** Estimate of disomes in log-phase wild type cells. To estimate the content of RNAse-resistant disomes in log-phase cells, the area below the A_{260} trace (fractions 6-10) of wild $type_{-glc}$ extract was subtracted from the area below the wild $type_{log/RNase}$ A_{260} trace (fractions 6-10) (Fig. 1a). Based on this approach we estimate that in the wild type about 7% of ribosomes had formed RNase-resistant disomes. The number agrees well with a previous estimate based on disome-seq data [6].

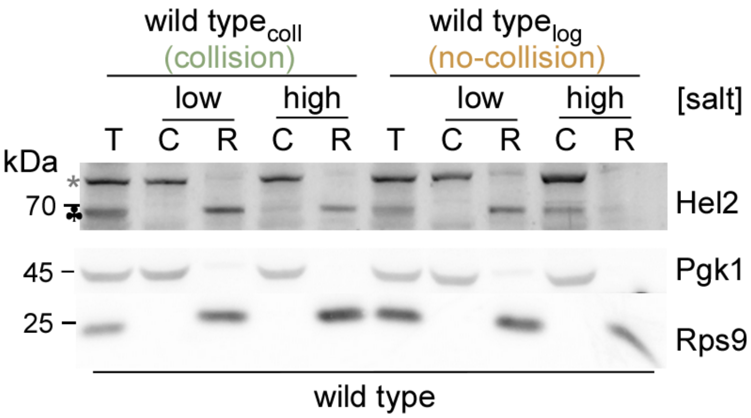

Figure S2. **Low-dose CHX treatment of log-phase cells induces salt-resistant ribosome-binding of Hel2.** Extracts were prepared from log-phase cells (no-collision, beige) or from log-phase cells after 10 min low-dose CHX treatment (collision, green). Ribosome-binding assays were performed as described in Fig. 2c and Methods. Aliquots of yeast extracts (T), cytosolic fractions (C), and ribosomal pellets (R) were analyzed by immunoblotting with α-Hel2, α-Pgk1 (cytosolic marker), and α-Rps9 (ribosomal marker). Statistic analysis is shown in Fig. 2d.

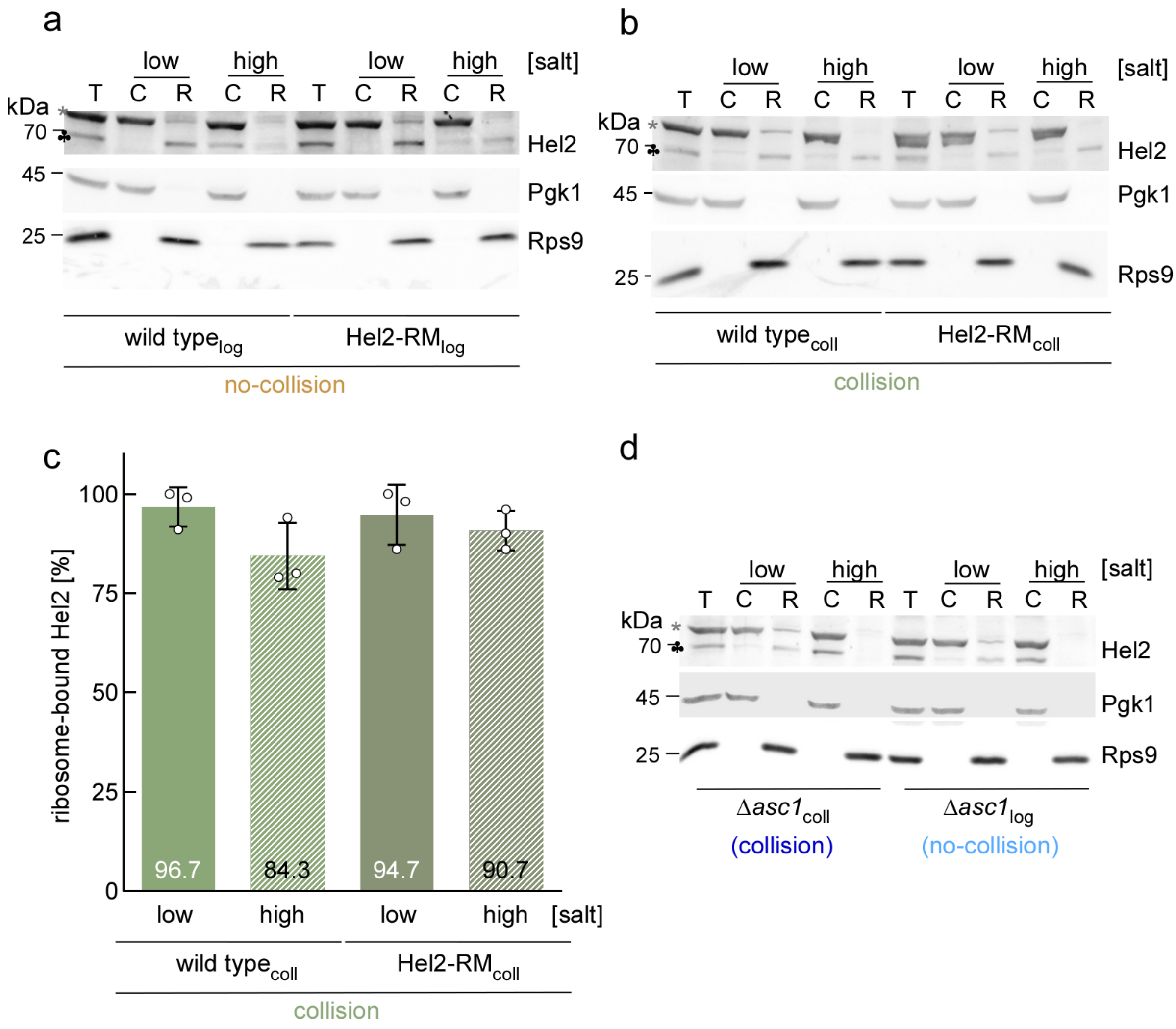

Figure S3. Ribosome-binding of Hel2 is affected by its catalytic activity and by the small ribosomal subunit protein Asc1. (**a, b, d**) Example blots of ribosome-binding assays shown in Fig. 3b, 3d, and S3c. Ribosome-binding assays were performed with extracts of Hel2$_{log}$, Hel2$_{coll}$, Hel2-RM$_{log}$, Hel2-RM$_{coll}$, Δ*asc1*$_{log}$, and Δ*asc1*$_{coll}$ as described in Fig. 2c and Methods. (T), cytosolic fractions (C), and ribosomal pellets (R), probed with α-Hel2, α-Pgk1 (cytosolic marker), and α-Rps9 (ribosomal marker). (**c**) Ribosome-binding of Hel2 and Hel2-RM after induction of collision. Shown is the mean of 3 independent experiments (bars) and the result of each experiment (dots). An example blot is shown in Fig. S3b.

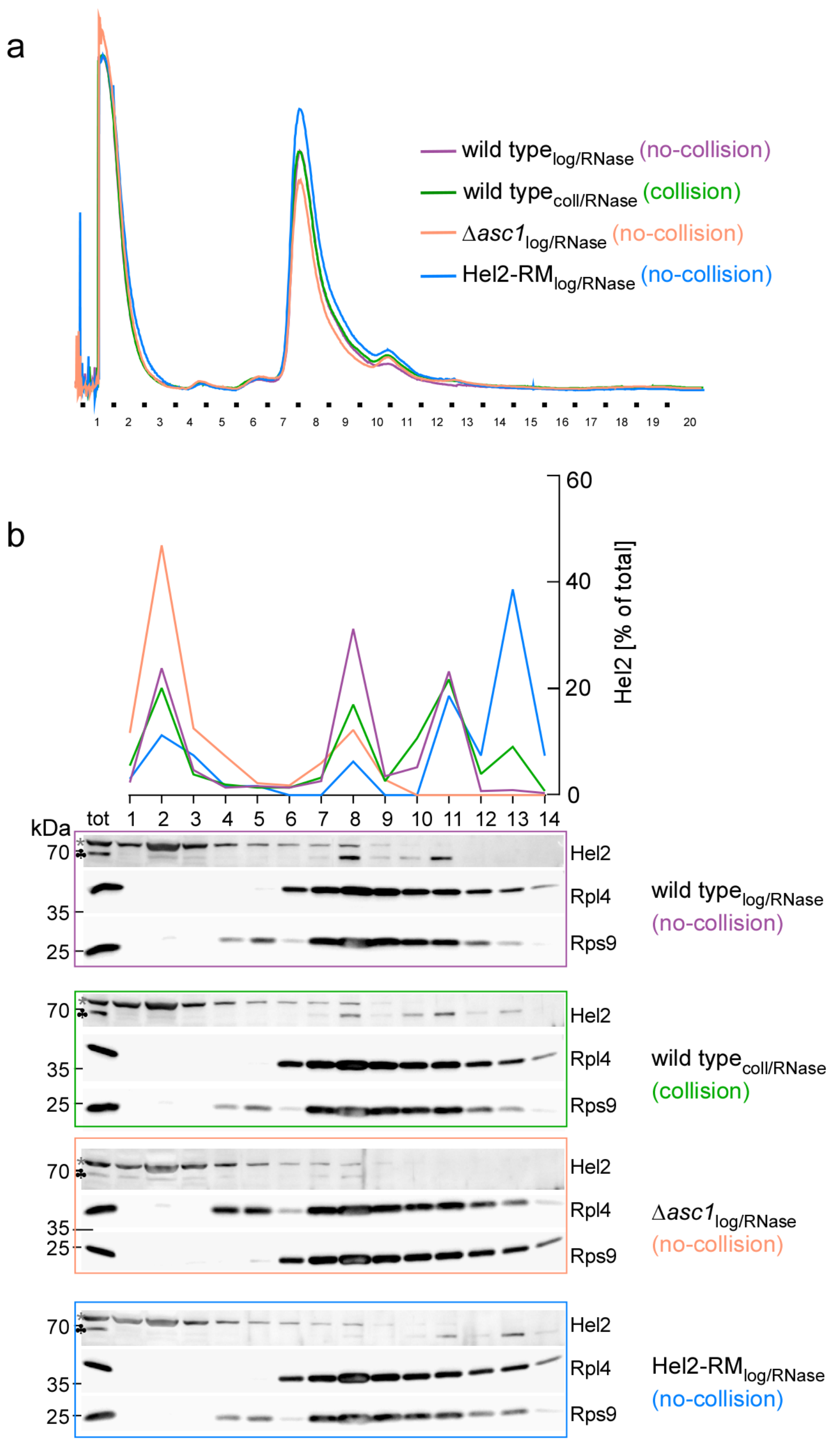

Figure S4. Distribution of Hel2 in ribosome profiles of RNase-treated extracts. (**a**) Overlay of complete A_{260} traces of wild type$_{log/RNase}$, wild type$_{coll/RNase}$, Δ*asc1*$_{log/RNase}$, and Hel2-RM$_{g\text{-}log/RNase}$ as shown in Fig. 4. (**b**) Hel2 distribution profiles (upper panel) and Hel2, Rpl4, and Rps9 immunoblots (lower panel) of fractions 1-14. The total (tot) corresponds to 5% of the extract loaded onto the gradient. To avoid overloading loading of fraction 2 was reduced to 50%.

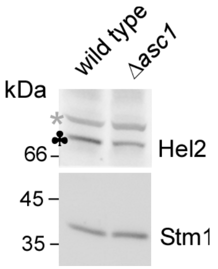

Figure S5. The steady state level of Hel2 is reduced in the Δ*asc1* strain. Example blot for the quantification and statistical analysis shown in Fig. 5a. Lysate of wild type or Δ*asc1* cells was analyzed by immunoblotting with α-Hel2 and α-Stm1 (loading control).

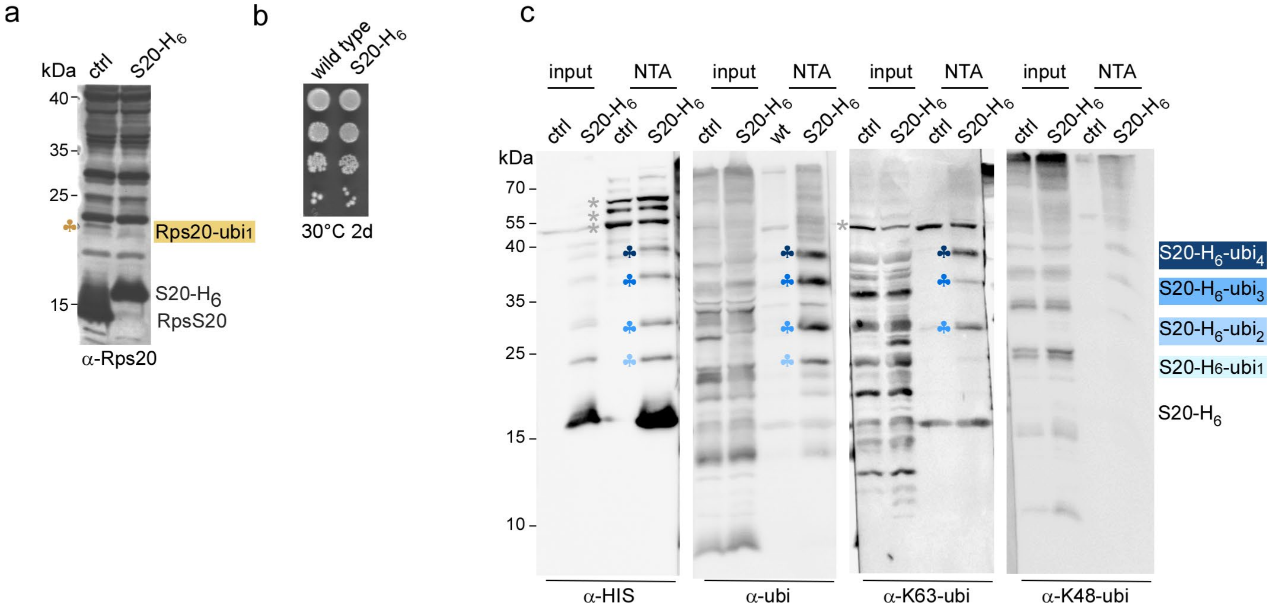

Figure S6. **Characterization of Rps20 polyubiquitination pattern in wild type cells.** (**a**) Lysate of wild type or S20-H$_6$ cells was analyzed by immunoblotting with α-Rps20. Rps20, S20-H$_6$, and Rps20-ubi$_1$ are indicated. S20-H$_6$-ubi$_1$ as well as more highly ubiquitinated Rps20/S20-H$_6$ species were concealed by strong cross-reactions of α-Rps20. (**b**) The S20-H$_6$ strain displays wild type growth at 30°C. Serial 10-fold dilutions of wild type and S20-H$_6$ strains were spotted onto YPD plates and were incubated at 30°C for 2 days. (**c**) Polyubiquitin chains attached to S20-H$_6$ are predominantly linked via K63. Preparation of yeast lysate and subsequent purification of S20-H$_6$ by Ni-NTA was performed with S20-H$_6$ and wild type cells (ctrl) under denaturing conditions as detailed in Methods. The input represents 5% of the material used for Ni-NTA (NTA) purification. Aliquots were analyzed by immunoblotting with α-HIS (detection of S20-H$_6$ and polyubiquitinated S20-H$_6$ species), α-ubi (detection of mono- and polyubiquitinated species of any kind), α-K63-ubi (detection of K63-linked ubiquitin chains), and α-K48-ubi (detection of K48-linked ubiquitin chains). Blue clubs indicate S20-ubi$_{1-4}$. Gray asterisks indicate background bands recognized by α-HIS.

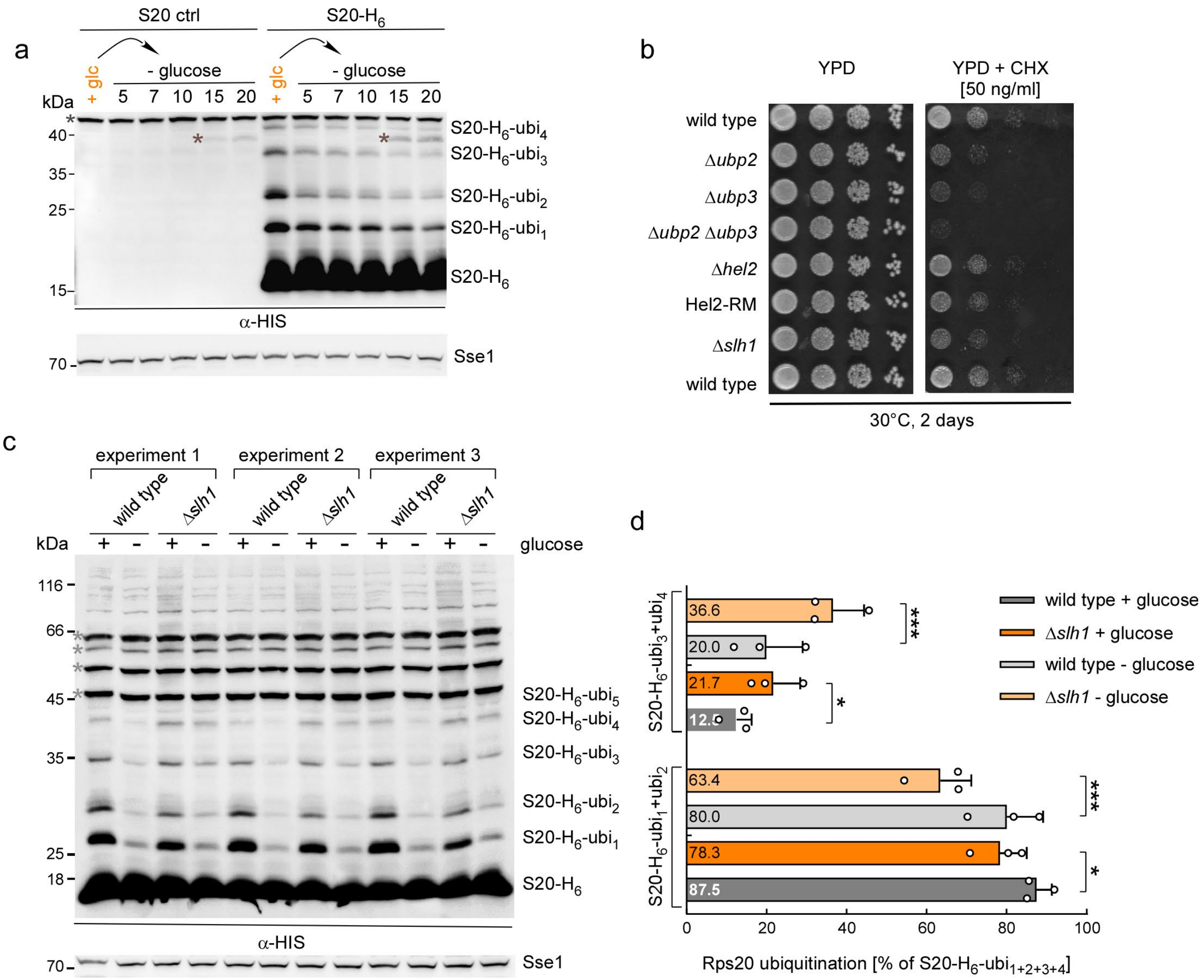

Figure S7. α-HIS detects an additional background band in glucose depleted cells. (**a**) Glucose depletion was performed with untagged wild type and S20-H_6 strains as described Fig. 7a. Samples were withdrawn at the time points indicated, shock-frozen and subsequently analyzed by immunoblotting with α-HIS. Sse1 served as loading control. Gray asterisks indicate background bands of α-HIS, brown asterisks indicate the additional background band of α-HIS, which appeared in glucose-depleted untagged wild type cells as well as in the S20-H_6 strain. (**b**) The Δ*ubp2*, Δ*ubp3*, and Δ*ubp2*Δ*ubp3* strains are sensitive towards cycloheximide. Serial 10-fold dilutions of the strains indicated were spotted onto YPD plates with or without 50 ng/ml cycloheximide (CHX) and were subsequently incubated at 30°C for 2 days. (**c**) Ubiquitination of S20-H_6 in the wild type and Δ*slh1* background. For details see Fig. 7c. The length of polyubiquitin chains (ubi_1 to ubi_{5+x}) is indicated. Shown are 3 independent experiments (1-3) used for analysis in Fig. 7f. **(d)** Statistical analysis of the experiment shown in **b**. S20-H_6-ubi_{1+2} represents the sum of mono- and di-ubiquitinated Rps20; S20-H_6-ubi_{3+4} represents the sum of tri- and tetra-ubiquitinated Rps20. Shown is percentage of S20-H_6-ubi_{1+2} and S20-H_6-ubi_{3+4} of the total S20-H_6-$ubi_{1+2+3+4}$. n.s.: $p > 0.05$, * p: < 0.05, **: $p < 0.01$, ***: $p < 0.001$.

SUPPLEMENTARY TABLES

Table S1. Plasmids.

plasmid	ori and marker	reference	#
pYCplac111	CEN, *LEU2*	[13]	257
pYEplac181	2µ, *LEU2*	[13]	5
pYEplac195	2µ, *URA3*	[13]	3
pYEplac195-Luc-R12$_{(CGA)}$-3xHA	2µ, *URA3*	this study	2278
pYEplac195-Luc-R12$_{(CGA)}$-Leu2	2µ, *URA3*	this study	2638
pYCplac111-Hel2-C64S	CEN, *LEU2*	this study	2029
pYEplac181-Hel2	2µ, *LEU2*	this study	2034
pYEplac181-Hel2-C64S	2µ, *LEU2*	this study	2506
pUG6	*kan*MX	[14]	608
pUG27	*HIS5*	[14]	609
pUG73	*LEU2*	[14]	612
pYM22	*TRP1*	Euroscarf [15]	1353
pYM46 (S20-H_6)	*kan*MX	Euroscarf [15]	1390
pRCC-N	*natMX*	pRCC-N was a gift from Eckhard Boles (Addgene plasmid #81192; http://n2t.net/addgene:81192; RRID:Addgene_81192)[16]	2632
pRCC-N (K6R, K8R, K6R-K8R)	*natMX*	this study	2752
pRCC-N-Rps20 PAM23	*natMX*	this study	2752

Table S2. Yeast strains.

strain	genotype	reference	#
MH272-3fα (wild type)	(*ura3*, *leu2*, *his3*, *trp1*, *ade2*)	[17]	6
Δ*hel2*	Δ*hel2::HIS5*	this study	3593
Hel2-RM_g	*HEL2*-C64S (genomic)	this study	5442
Hel2↑	Δ*hel2::HIS5* + pYEplac181-Hel2	this study	4099
Hel2-RM↑	Δ*hel2::HIS5* + pYEplac181-Hel2-C64S	this study	5048
Δ*asc1*	Δ*asc1::TRP1* (snoRNA intact)	[18]	3069
Δ*erg6*	Δ*erg6::HIS5*	this study	5387
Δ*asc1*Δ*erg6*	Δ*erg6::HIS5,* Δ*asc1::TRP1* (snoRNA intact)	this study	5389
Rps20-K6R	*RPS20*-K6R (genomic)	this study	5619
Rps20-K8R	*RPS20*-K8R (genomic)	this study	5620
Rps20-K6R-K8R	*RPS20*-K6R-K8R (genomic)	this study	5621
S20-H_6	*RPS20-HIS*$_6$ - *KanMX4* (genomic)	this study	3599
S20-H_6-K6R	S20-H_6-K6R (genomic)	this study	5616
S20-H_6 K8R	S20-H_6-K8R (genomic)	this study	5617
S20-H_6 K6R K8R	S20-H_6K6R-K8R (genomic))	this study	5618
S20-H_6 Δ*hel2*	S20-H_6 Δ*hel2::HIS5*	this study	3815
S20-H_6 Hel2↑	S20-H_6 Δ*hel2::HIS5* + pYEplac181-Hel2	this study	4151
S20-H_6 Hel2-RM↑	S20-H_6 Δ*hel2::HIS5* + pYEplac181-Hel2-C64S	this study	5597
S20-H_6 Δ*asc1*	S20-H_6 Δ*asc1::TRP1*	this study	3986
Δ*ubp2*	Δ*ubp2::LEU2*	this study	4758
Δ*ubp3*	Δ*ubp3::HIS5*	this study	4759
Δ*ubp2*Δ*ubp3*	Δ*ubp2::LEU2* Δ*ubp3::HIS5*	this study	4765
Δ*slh1*	Δ*slh1::TRP1*	this study	5500
S20-H_6 Δ*ubp2*	S20-H_6 Δ*ubp2::LEU2*	this study	4369
S20-H_6 Δ*ubp3*	S20-H_6 Δ*ubp3::HIS5*	this study	4257
S20-H_6 Δ*ubp2*Δ*ubp3*	S20-H_6 Δ*ubp2::LEU2* Δ*ubp3::HIS5*	this study	4370
S20-H_6 Δ*slh1*	S20-H_6 Δ*slh1::TRP1*	this study	5323

Table S3. Primers.

primer	5´-3´ sequence	#
**plasmid construction**		
HindIII-Hel2-F	TTCCGTGCTTAAGCTTAAGCAAGAGTACAATTCAGGAACTC	27019
PstI-Hel2-R	TTCCGTGCTTCTGCAGCTTTTAGCATTGTCCACAGGC	27020
Hel2-C64S-F	GATGAAGAAAATGAATTATCTGTAATTTGTGCGCGCAAG	27041
Hel2-C64S-R	CTTGCGCGCACAAATTACAGATAATTCATTTTCTTCATC	27042
PstI-Leu2-F	TTCCGTGCTTCTGCAGTCTGCCCCTAAGAAGATCGTC	39021
XhoI-Leu2-R	TTCCGTGCTTCTCGAGGTATTCTTGCCACGACTCATCTC	39022
UP-F (Hel2 + 50bp) (pYCplac111-**Hel2-C64S**)	AGTCTCTTTCGTCGAAAAAATAGTG	39067
DOWN-R (Hel2 - 54bp) (pYCplac111-**Hel2-C64S**)	GACTTTCATTTCTCTAATGCTATTGTCAG	39068
Seq-Hel2-F1	ATGAGCGAATCAGTGAAAGAAAAC	27045
Hel2-1014bp-Rev	ATCATCTCTTTCCCTGATCACTC	39028
**amplification of deletion cassettes**		
GD-hel2-FW (*hel2*::*HIS5*)	AGTCTCTTTCGTCGAAAAAATAGTGGCTATACTTCTTTTCAAGAATTAGGcagctgaagc ttcgtacgc	26070
GD-hel2-Rev (*hel2*::*HIS5*)	TTCATTTCTCTAATGCTATTGTCAGTTACAGGTTAGAAATATATTTCCAAgcataggcca ctagtggatctg	26071
GD-erg6-FW (*erg6*::*HIS5*)	CATAATTTAAAAAAACAAGAATAAAATAATAATATAGTAGGCAGCATAAGcagctgaagc ttcgtacg	39008
GD-erg6-Rev (*erg6*::*HIS5*)	AAATAGGTATATATCGTGCGCTTTATTTGAATCTTATTGATCTAGTGAATgcataggcca ctagtggatctg	39009
GD-ubp2-FW (*ubp2*::*LEU2*)	GTGGTAATTAAAAAGAAAGCTTTTGTTCAAGGTTAAGAAGGTATAAGGAAcagctgaagc ttcgtacgc	31048
GD-ubp2-Rev (*ubp2*::*LEU2*)	GGTACTTATGGCAATAGTGACATTTTACATAAACTCTTCATTGACTAAGAgcataggcca ctagtggatctg	31049
GD-ubp3-FW (*ubp3*::*HIS5*)	CTGCTACCATCATCCAGGTACCGCTTTCCTTTCCATCATCATTAAAAAAAcagctgaagc ttcgtacgc	31050
GD-ubp3-Rev (*ubp3*::*HIS5*)	TATTGCTATATTATTTTTTATGTATTTTGTCTATAATACCACCCCCCGTCgcataggcca ctagtggatctg	31051
GD-slh1-TRP1 F (*slh1*::*TRP1*)	ACCATTGGAATTGTGAGAAGTAGATCCGTACCATCAATAGCCGGCTCAAGgcaaagtgga acgatcattcac	38051

**epitope tagging**		
Rps20-6HIS-FW (*RPS20*-HIS_6 - KanMX4)	CAAATCACCATTGAACCTGGTGTGGATGTCGAAGTTGTTGTTGCTTCCAACAGCAGCGGT caccatcaccatcaccattag	26079
Rps20-6HIS-Rev (*RPS20*-HIS_6 - KanMX4)	TGGACGAGAAAAAGAGGAATCTAATGGAAATTATTTCCAGTTAGAACCAGCatcgatgaa ttcgagctcg	26080
UBP2-CT-F (*UBP2*-3xHA – kl*TRP1*)	TCAAACAAGGACAAGAAGGTGATATTGAGCCATTGAAAAGAATTCTAAAGcgtacgctgc aggtcgac	33070
UBP2-CT-R (*UBP2*-3xHA – kl*TRP1*)	GGTACTTATGGCAATAGTGACATTTTACATAAACTCTTCATTGACTAAGAatcgatgaat tcgagctcg	33071
**CRISPR-Cas9 genome editing**		
WGP235-pCC2-Fw (pRCC-Nat-Rps20 PAM23)	TGTTGTCTGACATTTTGAGAGTTAACACCGAAATTACCAAGGCTC	39066
WGP234-pCC1-Rv (pRCC-Nat-Rps20 PAM23)	CTTGGTGGTGTTCGTCGTATCTCTTAATCATAGAAGCAGACAATGGAG	39065
CC_Rps20 PAM23-Fw	GTCTGACTTTCAAAAGGAAAGTTTTAGAGCTAGAAATAGCAAGTTAAAATAAGG	41076
CC_Rps20 PAM23-Rev	TTTCCTTTTGAAAGTCAGACGATCATTTATCTTTCACTGCGGAG	41077
Donor DNA (Rps20 K6R)	GTAAAATAAACAAAAAGGTATATACAAAATGTCTGACTTTCAAAGAGAAAAAGTTGAAGA ACAAGAACAACAACAACAACAAATCATCAAGATTAGAATC	41078
Donor DNA (Rps20 K8R)	GTAAAATAAACAAAAAGGTATATACAAAATGTCTGACTTTCAAAAAGAAAGAGTTGAAGA ACAAGAACAACAACAACAACAAATCATCAAGATTAGAATC	41079
Donor DNA (Rps20 K6R-K8R)	GTAAAATAAACAAAAAGGTATATACAAAATGTCTGACTTTCAAAGAGAAAGAGTTGAAGA ACAAGAACAACAACAACAACAAATCATCAAGATTAGAATC	41080

Supplementary Data 1. Quantification of ribosomal complexes and Hel2 occupancy (xlsx file). Related to Fig. 4. Calculation was performed with two independent data sets. The quantification of experiment 2 is shown in Fig. 4e.

Supplementary Data 2. Original blots employed for statistical analysis (xlsx file).

SUPPLEMENTARY REFERENCES

1. Guydosh, N. R. & Green, R. Dom34 rescues ribosomes in 3' untranslated regions. *Cell* **156,** 950-962 (2014).
2. Diament, A. *et al.* The extent of ribosome queuing in budding yeast. *PLoS Comput. Biol.* **14,** e1005951 (2018).
3. Juszkiewicz, S. *et al.* ZNF598 Is a Quality Control Sensor of Collided Ribosomes. *Mol. Cell* **72,** 469-481 e467 (2018).
4. Ikeuchi, K. *et al.* Collided ribosomes form a unique structural interface to induce Hel2-driven quality control pathways. *EMBO J.* **38** (2019).
5. Matsuo, Y. & Inada, T. The ribosome collision sensor Hel2 functions as preventive quality control in the secretory pathway. *Cell Rep.* **34,** 108877 (2021).
6. Zhao, T. *et al.* Disome-seq reveals widespread ribosome collisions that promote cotranslational protein folding. *Genome Biol.* **22,** 16 (2021).
7. Sinha, N. K. *et al.* EDF1 coordinates cellular responses to ribosome collisions. *Elife* **9** (2020).
8. Stansfield, I., Grant, G. M., Akhmaloka & Tuite, M. F. Ribosomal association of the yeast SAL4 (SUP45) gene product: implications for its role in translation fidelity and termination. *Mol. Microbiol.* **6,** 3469-3478 (1992).
9. Ashe, M. P., De Long, S. K. & Sachs, A. B. Glucose depletion rapidly inhibits translation initiation in yeast. *Mol. Biol. Cell* **11,** 833-848 (2000).
10. Gilbert, W. V., Zhou, K., Butler, T. K. & Doudna, J. A. Cap-independent translation is required for starvation-induced differentiation in yeast. *Science* **317,** 1224-1227 (2007).
11. Raue, U., Oellerer, S. & Rospert, S. Association of protein biogenesis factors at the yeast ribosomal tunnel exit is affected by the translational status and nascent polypeptide sequence. *J. Biol. Chem.* **282,** 7809-7816 (2007).
12. Janapala, Y., Preiss, T. & Shirokikh, N. E. Control of Translation at the Initiation Phase During Glucose Starvation in Yeast. *Int. J. Mol. Sci.* **20** (2019).
13. Gietz, R. D. & Sugino, A. New yeast-*Escherichia coli* shuttle vectors constructed with in vitro mutagenized yeast genes lacking six-base pair restriction sites. *Gene* **74,** 527-534 (1988).
14. Gueldener, U., Heinisch, J., Koehler, G. J., Voss, D. & Hegemann, J. H. A second set of loxP marker cassettes for Cre-mediated multiple gene knockouts in budding yeast. *Nucleic Acids Res.* **30,** e23 (2002).
15. Janke, C. *et al.* A versatile toolbox for PCR-based tagging of yeast genes: new fluorescent proteins, more markers and promoter substitution cassettes. *Yeast* **21,** 947-962 (2004).
16. Generoso, W. C., Gottardi, M., Oreb, M. & Boles, E. Simplified CRISPR-Cas genome editing for Saccharomyces cerevisiae. *J. Microbiol. Methods* **127,** 203-205 (2016).
17. Heitman, J., Movva, N. R., Hiestand, P. C. & Hall, M. N. FK 506-binding protein proline rotamase is a target for the immunosuppressive agent FK 506 in Saccharomyces cerevisiae. *Proc. Natl. Acad. Sci. U S A* **88,** 1948-1952 (1991).
18. Chiabudini, M. *et al.* Release factor eRF3 mediates premature translation termination on polylysine-stalled ribosomes in Saccharomyces cerevisiae. *Mol. Cell. Biol.* **34,** 4062-4076 (2014).

Uncropped blots Figures 1-7

Fig. 1a

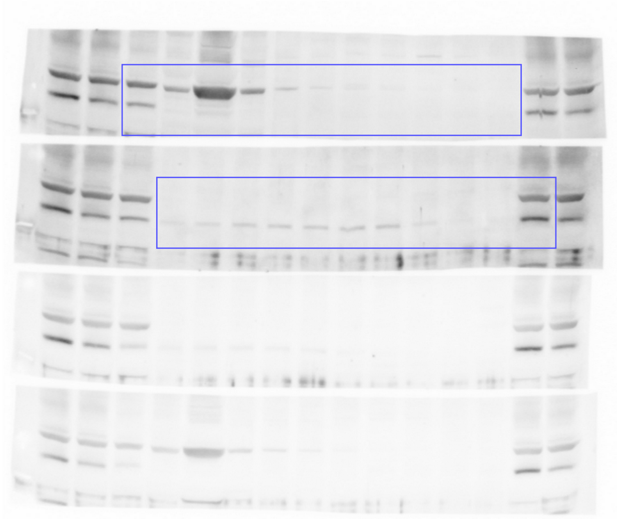

Hel2

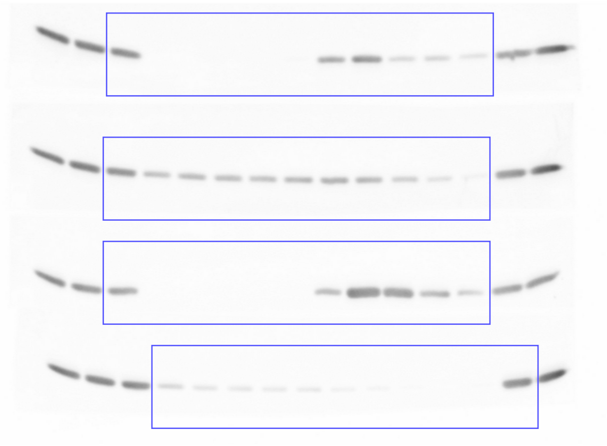

Rpl4

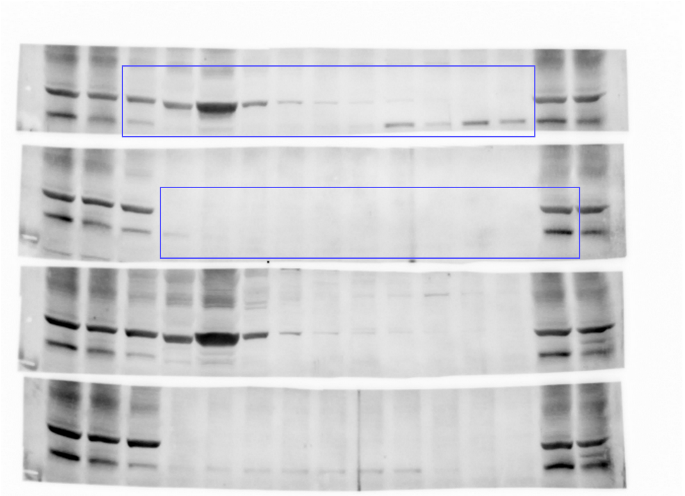

Hel2

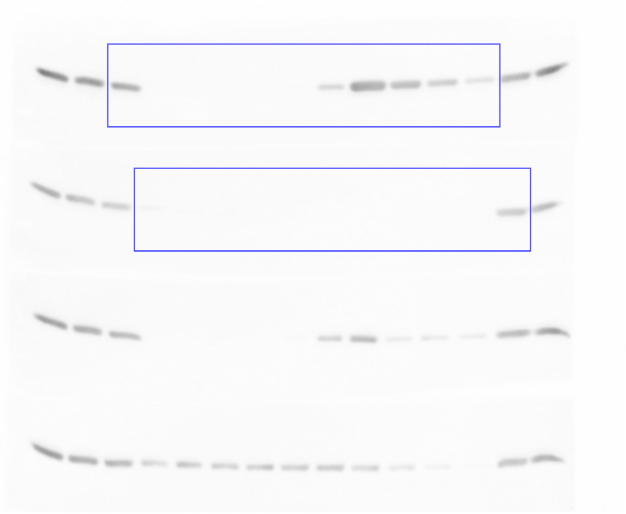

Rpl4

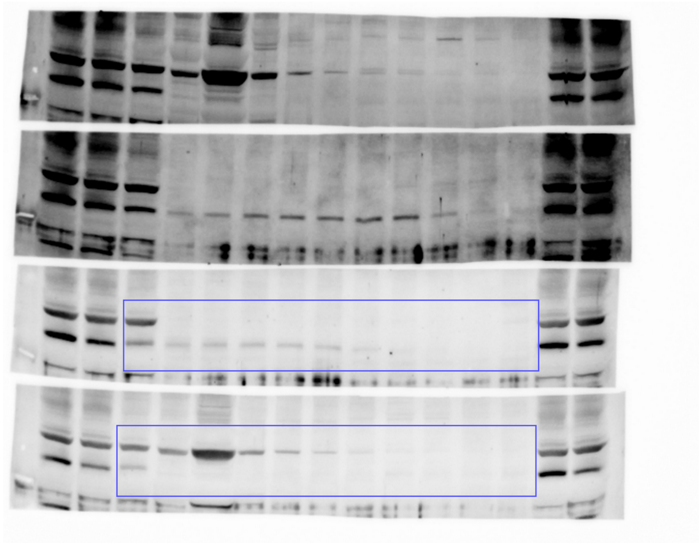

Hel2

Fig. 1b

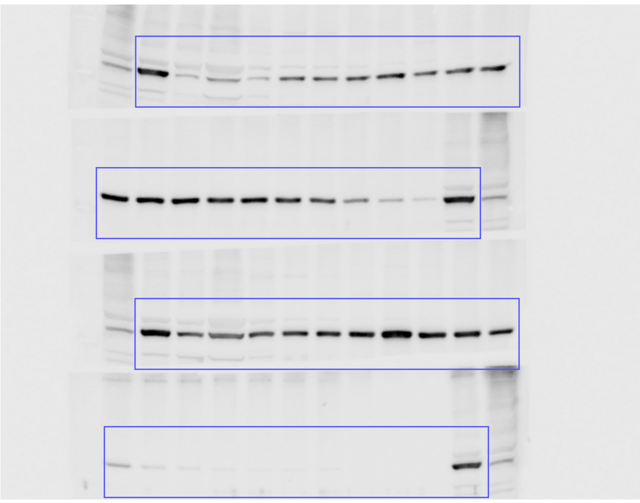

Hel2

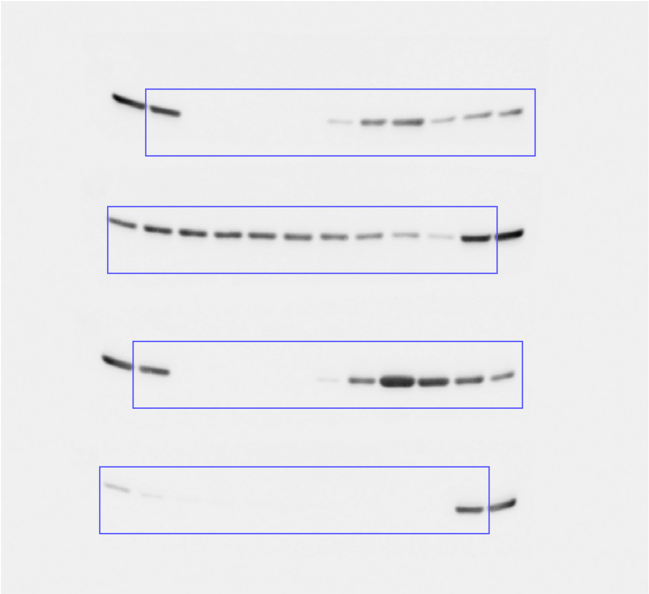

Rpl4

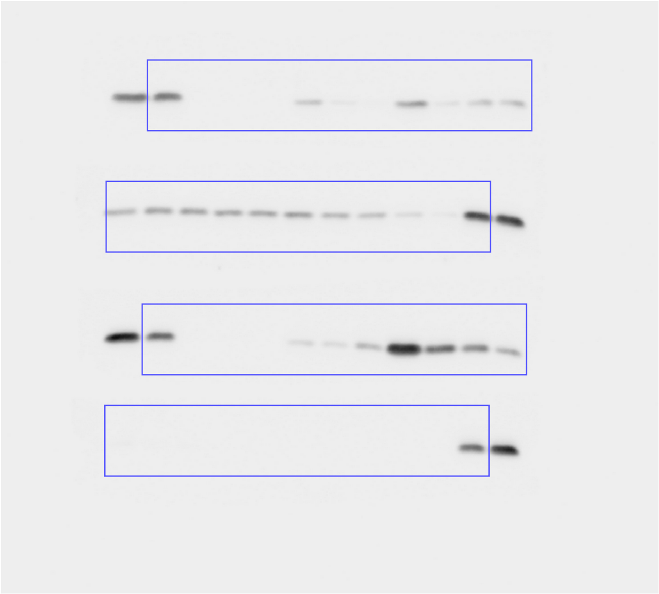

Rps9

Fig. 1c

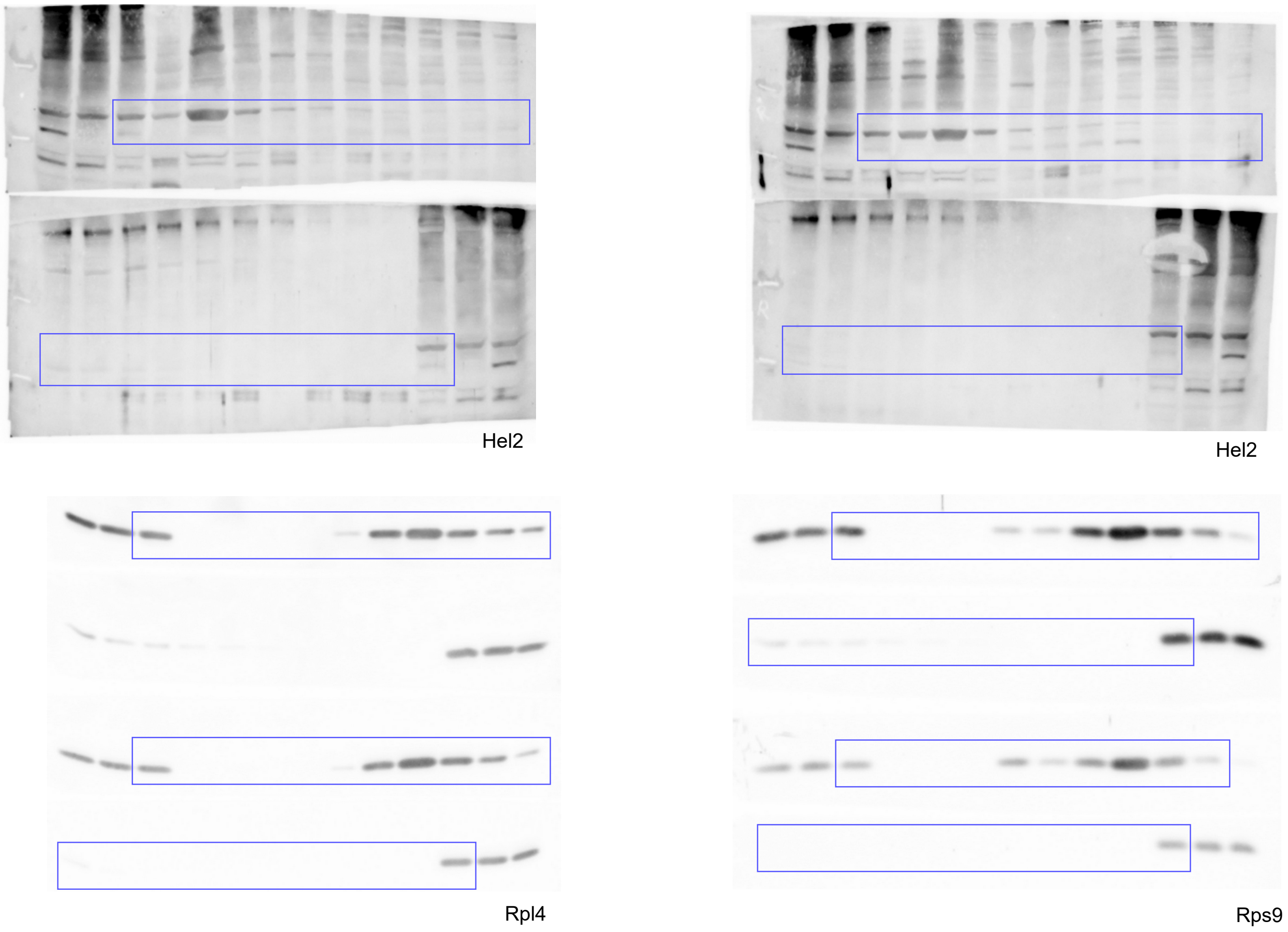

Fig. 2a

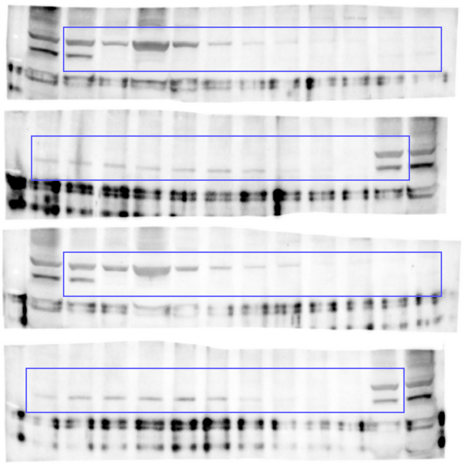

Hel2

Fig. 2b

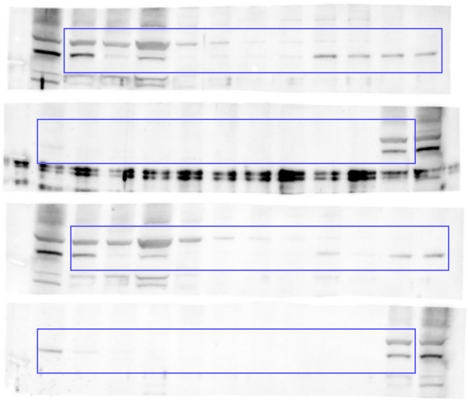

Hel2

Fig. 3a

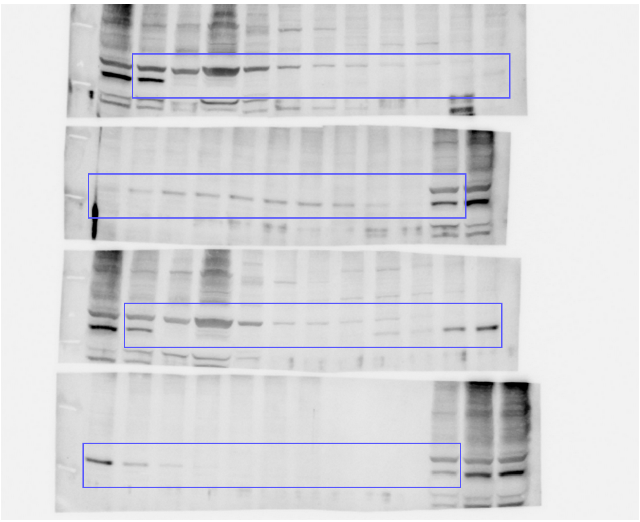

Hel2

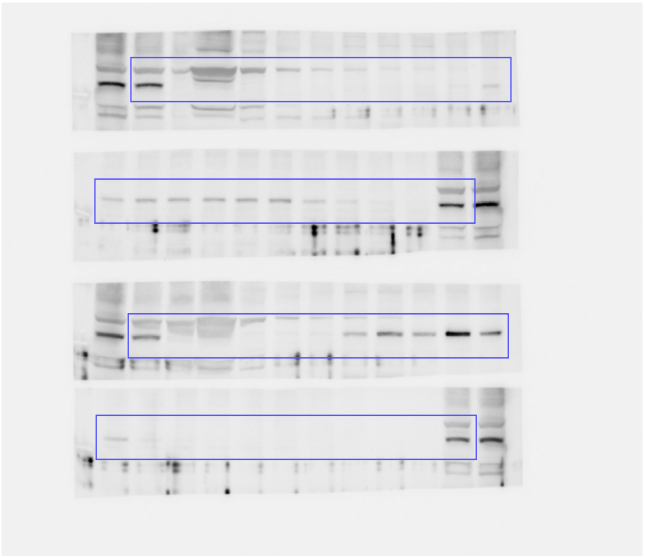

Hel2

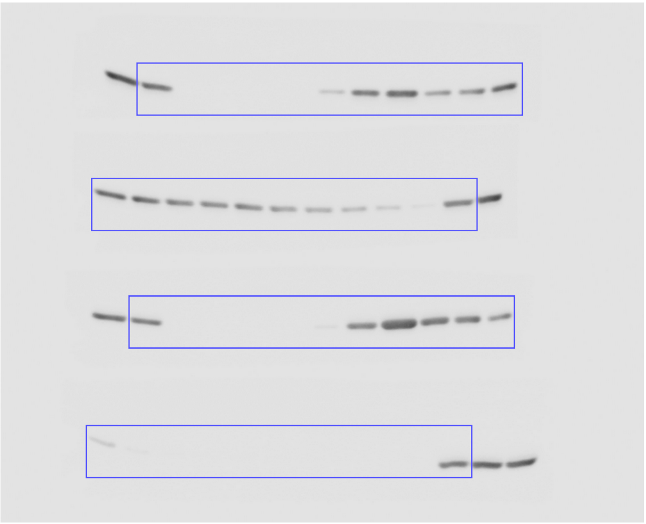

Rpl4

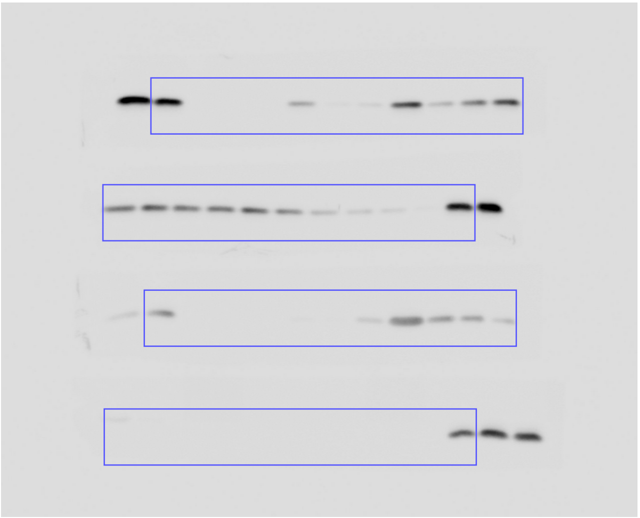

Rps9

Fig. 3c

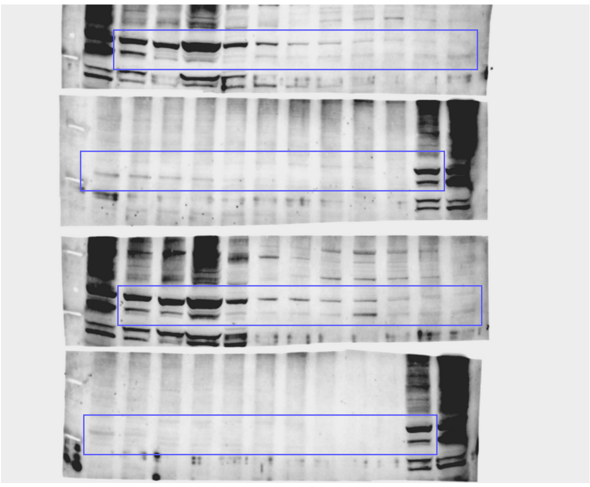

Hel2

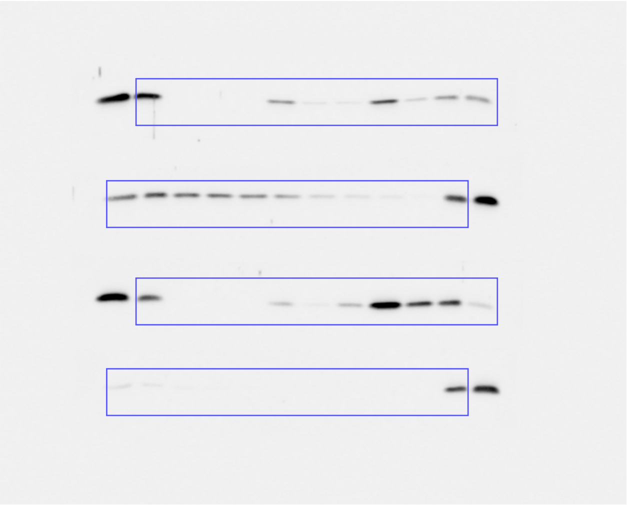

Rps9

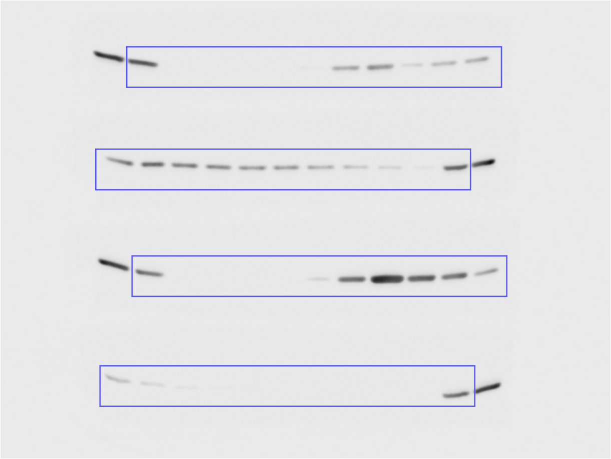

Rpl4

Fig. 4a-d

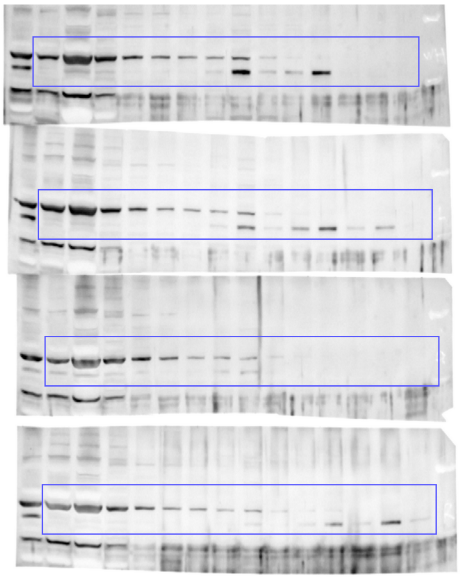

Hel2

Fig. 5b

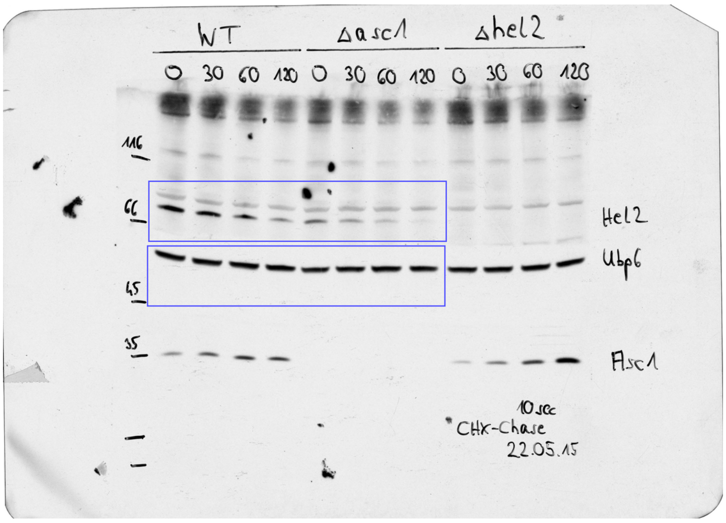

Fig. 5d

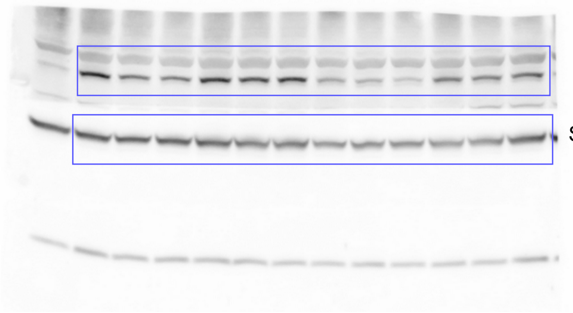

Hel2

Stm1

Fig. 6a

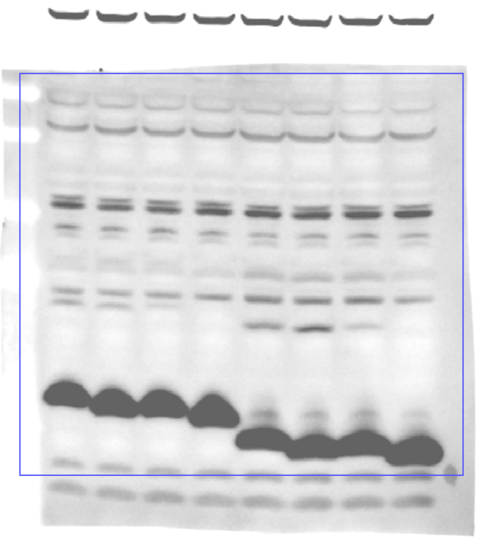

S20-H$_6$

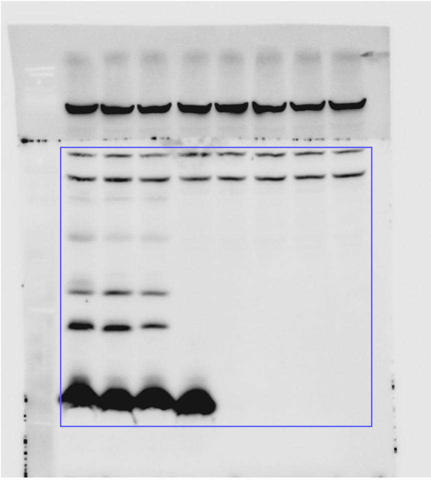

S20-H$_6$

Fig. 6b

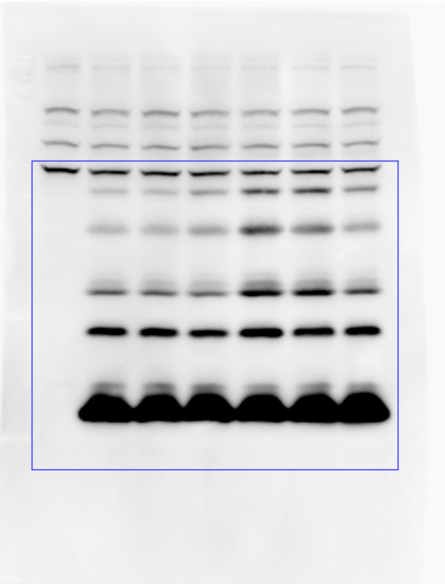

S20-H$_6$

Fig. 6c

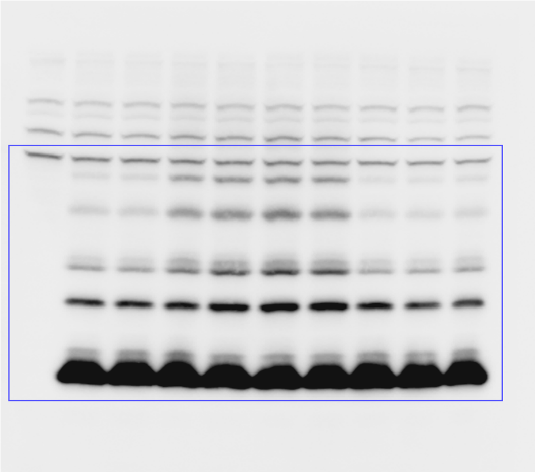

S20-H$_6$

Fig. 6d

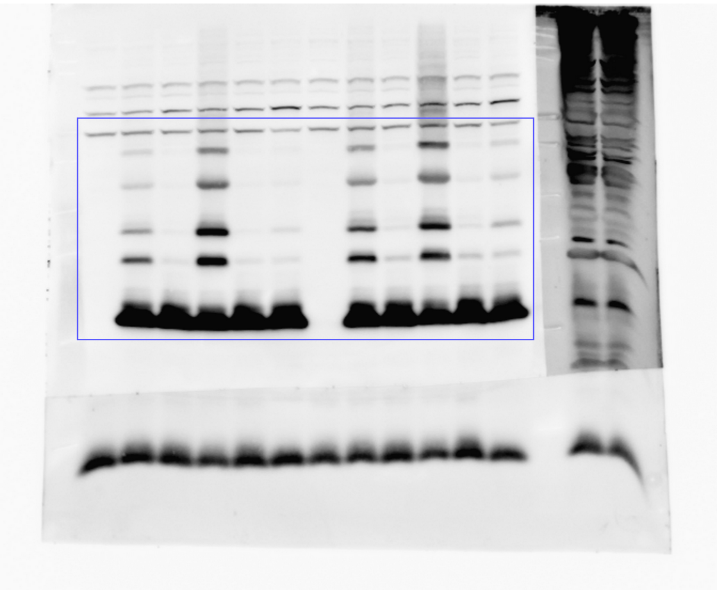

S20-H$_6$

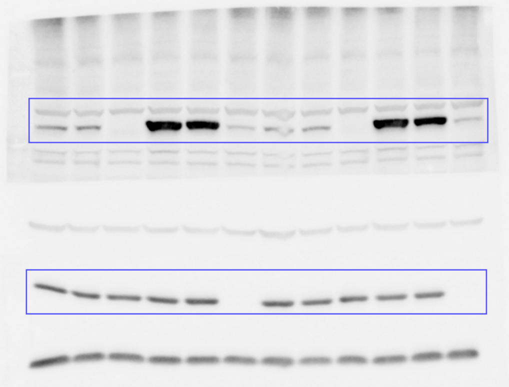

Hel2

Asc1

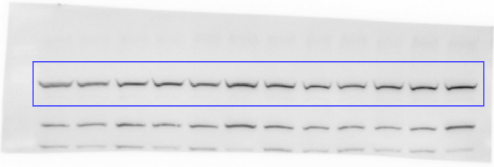

Sse1

Fig. 7a

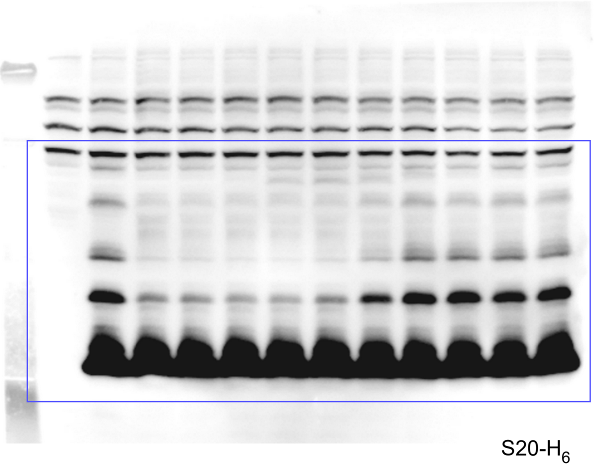

S20-H_6

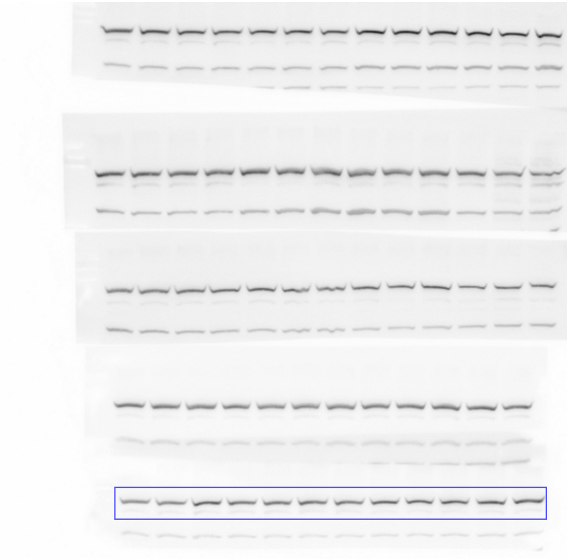

Sse1

Fig. 7b

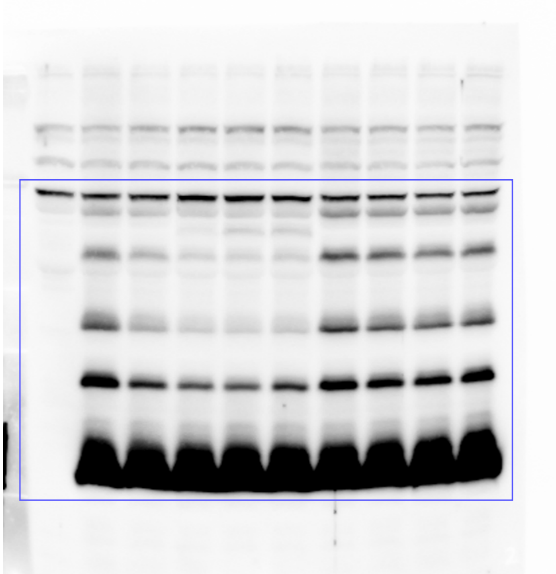

S20-H_6

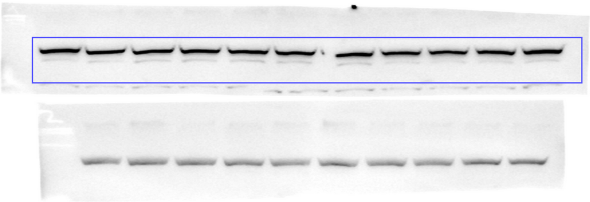

Sse1

Fig. 7c

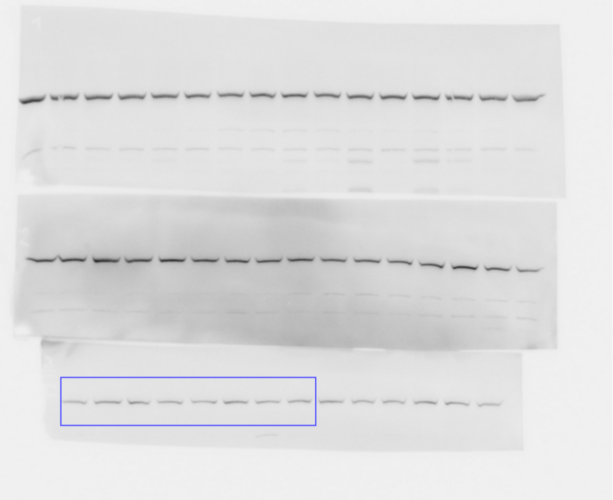

Sse1

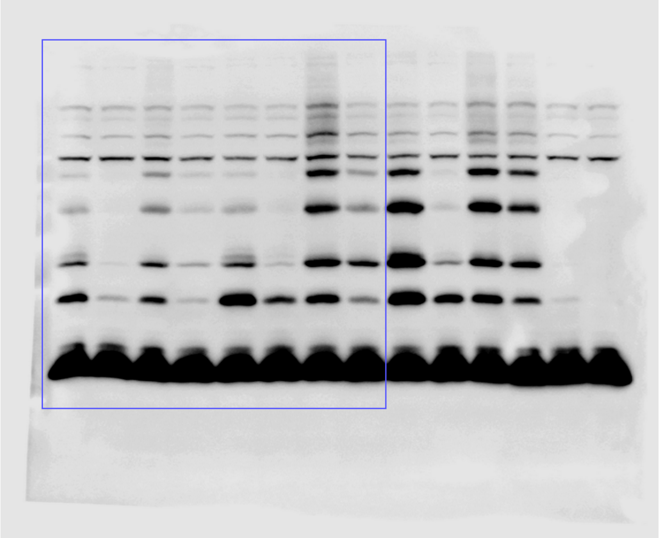

S20-H_6

Fig. 7e

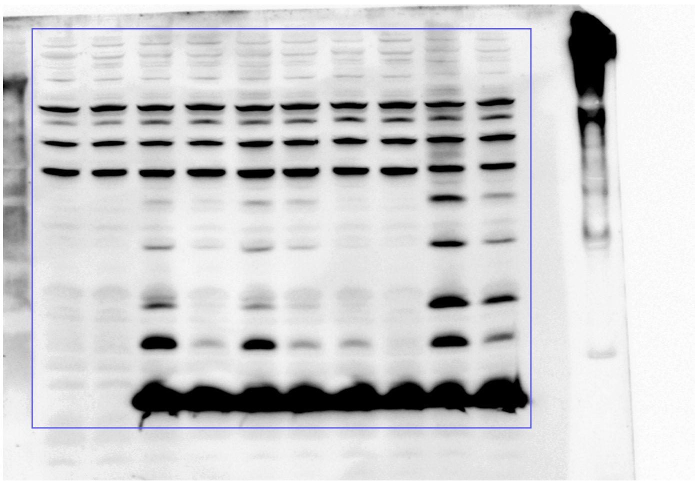

$S20\text{-}H_6$

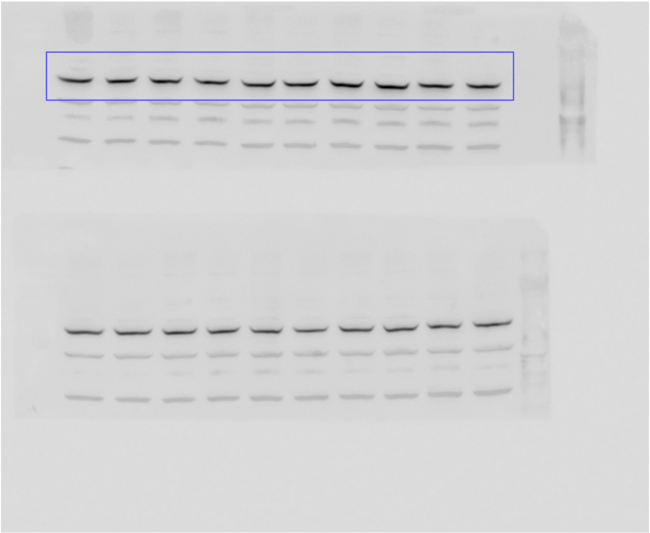

Sse1
