# Supplementary material for: Stalled disomes marked by Hel2-dependent ubiquitin chains undergo Ubp2/Ubp3-mediated deubiquitination upon translational run-off
Source: Commun Biol. 2025 Jan 28;8:132. doi: 10.1038/s42003-025-07569-z (PMC11775340; doi:10.1038/s42003-025-07569-z)
Supplement: Supplementary file 2 — Description of Additional Supplementary Files [file 42003_2025_7569_MOESM2_ESM.pdf]

Description of Additional Supplementary Files

File name: Supplementary Data 1

Description: Quantification of ribosomal complexes and Hel2 occupancy (xlsx file). Related to Fig. 4. Calculation was performed with two independent data sets. The quantification of experiment 2 is shown in Fig. 4e.

File name: Supplementary Data 2

Description: Original blots employed for statistical analysis (xlsx file).
