# Supplementary material for: Stalled disomes marked by Hel2-dependent ubiquitin chains undergo Ubp2/Ubp3-mediated deubiquitination upon translational run-off
Source: Commun Biol. 2025 Jan 28;8:132. doi: 10.1038/s42003-025-07569-z (PMC11775340; doi:10.1038/s42003-025-07569-z)
Supplement: Supplementary file 5 — nr-reporting-summary [file 42003_2025_7569_MOESM5_ESM.pdf]

nature portfolio

Corresponding author(s): Sabine Rospert

Last updated by author(s): 20.12.2024

Reporting Summary

Nature Portfolio wishes to improve the reproducibility of the work that we publish. This form provides structure for consistency and transparency in reporting. For further information on Nature Portfolio policies, see our Editorial Policies and the Editorial Policy Checklist.

Statistics

For all statistical analyses, confirm that the following items are present in the figure legend, table legend, main text, or Methods section.

n/a	Confirmed	
☐	☒	The exact sample size (*n*) for each experimental group/condition, given as a discrete number and unit of measurement
☐	☒	A statement on whether measurements were taken from distinct samples or whether the same sample was measured repeatedly
☐	☒	The statistical test(s) used AND whether they are one- or two-sided<br>*Only common tests should be described solely by name; describe more complex techniques in the Methods section.*
☒	☐	A description of all covariates tested
☐	☒	A description of any assumptions or corrections, such as tests of normality and adjustment for multiple comparisons
☐	☒	A full description of the statistical parameters including central tendency (e.g. means) or other basic estimates (e.g. regression coefficient) AND variation (e.g. standard deviation) or associated estimates of uncertainty (e.g. confidence intervals)
☐	☒	For null hypothesis testing, the test statistic (e.g. *F*, *t*, *r*) with confidence intervals, effect sizes, degrees of freedom and *P* value noted<br>*Give P values as exact values whenever suitable.*
☒	☐	For Bayesian analysis, information on the choice of priors and Markov chain Monte Carlo settings
☒	☐	For hierarchical and complex designs, identification of the appropriate level for tests and full reporting of outcomes
☒	☐	Estimates of effect sizes (e.g. Cohen's *d*, Pearson's *r*), indicating how they were calculated

Our web collection on statistics for biologists contains articles on many of the points above.

Software and code

Policy information about availability of computer code

Data collection	no unpublished software was used
Data analysis	GraphPad Prism 10.1.1, Image J 1.54g

For manuscripts utilizing custom algorithms or software that are central to the research but not yet described in published literature, software must be made available to editors and reviewers. We strongly encourage code deposition in a community repository (e.g. GitHub). See the Nature Portfolio guidelines for submitting code & software for further information.

Data

Policy information about availability of data

All manuscripts must include a data availability statement. This statement should provide the following information, where applicable:

- Accession codes, unique identifiers, or web links for publicly available datasets
- A description of any restrictions on data availability
- For clinical datasets or third party data, please ensure that the statement adheres to our policy

All data supporting the findings of this study are available within the paper and its Supplementary Information.

Research involving human participants, their data, or biological material

Policy information about studies with human participants or human data. See also policy information about sex, gender (identity/presentation), and sexual orientation and race, ethnicity and racism.

Reporting on sex and gender	n/a
Reporting on race, ethnicity, or other socially relevant groupings	n/a
Population characteristics	n/a
Recruitment	n/a
Ethics oversight	n/a

Note that full information on the approval of the study protocol must also be provided in the manuscript.

Field-specific reporting

Please select the one below that is the best fit for your research. If you are not sure, read the appropriate sections before making your selection.

☒ Life sciences ☐ Behavioural & social sciences ☐ Ecological, evolutionary & environmental sciences

For a reference copy of the document with all sections, see nature.com/documents/nr-reporting-summary-flat.pdf

Life sciences study design

All studies must disclose on these points even when the disclosure is negative.

Sample size	Sample-size determination by statistical methods was not performed. Sample-size was adjusted for each set of experiments to reveal reproducibility and/or statistical significance of the data.
Data exclusions	No data were excluded from the analysis.
Replication	Reproducibility was verified by replications and statistical analysis of the data as indicated in the Figure Legends.
Randomization	Randomization is not applicable.
Blinding	Blinding is not applicable.

Reporting for specific materials, systems and methods

We require information from authors about some types of materials, experimental systems and methods used in many studies. Here, indicate whether each material, system or method listed is relevant to your study. If you are not sure if a list item applies to your research, read the appropriate section before selecting a response.

Materials & experimental systems

n/a	Involved in the study	
☐	☒	Antibodies
☒	☐	Eukaryotic cell lines
☒	☐	Palaeontology and archaeology
☒	☐	Animals and other organisms
☒	☐	Clinical data
☒	☐	Dual use research of concern
☒	☐	Plants

Methods

n/a	Involved in the study	
☒	☐	ChIP-seq
☒	☐	Flow cytometry
☒	☐	MRI-based neuroimaging

Antibodies

Antibodies used	Polyclonal antibodies Rospert lab collection: anti-Hel2 (Fig. S1b), anti-Rps20 (Fig. 5a), anti-Ubp6 (Fig. S1b), anti-Stm1 (Fig. S1b), anti-Asc1 (Fig. S1b and Gribling-Burrer et al, 2019), Rpl4 (Zhang et al, 2016), anti-Rps9 (Raue et al, 2007), anti-Sse1 (Raue et al, 2007), anti-Pgk1 (Zhang et al, 2020). Monoclonal antibodies: anti-HIS (BioRad, 620-0203), anti-ubiquitin P4D1 (Santa Cruz Biotechnology,

sc-8017), anti-K63-Ub clone Apu3 (Sigma-Aldrich, 05-1308), anti-K48-Ub D9D5 (Cell Signaling Technology 8081), anti-FLAG (Agilent 200474), anit-HA (Santa Cruz, Y-11, sc-805), anti-Luciferase (Sigma L0159), anti-rabbit HRP (Pierce, 61-6520) and anti-mouse HRP (Santa Cruz, sc-2748).

Validation	Antibodies directed against yeast proteins were validated by Western blotting. To that end, wild type and either deletions strains or (in case of essential genes) strains expressing a tagged-version of the respective protein were analyzed side-by-side. Anti-FLAG and anti-His were validated by comparing Western blots of wild type yeast and yeast strains expressing FLAG- or His-tagged proteins.

Plants

Seed stocks	n/a
Novel plant genotypes	n/a
Authentication	n/a
